# COMADRE: a global data base of animal demography

**DOI:** 10.1111/1365-2656.12482

**Published:** 2016-01-27

**Authors:** Roberto Salguero‐Gómez, Owen R. Jones, C. Ruth Archer, Christoph Bein, Hendrik de Buhr, Claudia Farack, Fränce Gottschalk, Alexander Hartmann, Anne Henning, Gabriel Hoppe, Gesa Römer, Tara Ruoff, Veronika Sommer, Julia Wille, Jakob Voigt, Stefan Zeh, Dirk Vieregg, Yvonne M. Buckley, Judy Che‐Castaldo, David Hodgson, Alexander Scheuerlein, Hal Caswell, James W. Vaupel

**Affiliations:** ^1^ Laboratory of Evolutionary Biodemography LaboratoryMax Planck Institute for Demographic ResearchKonrad‐Zuse‐Straße 1RostockDE‐18057Germany; ^2^ ARC Centre of Excellence for Environmental DecisionsSchool of Biological SciencesThe University of QueenslandGoddard building #8St. LuciaQld4072Australia; ^3^ Max‐Planck Odense Center on the Biodemography of AgingUniversity of Southern DenmarkOdenseDenmark; ^4^ Department of BiologyUniversity of Southern DenmarkOdenseDenmark; ^5^ MaxNetAging SchoolMax Planck Institute for Demographic ResearchKonrad‐Zuse‐Straße 1DE‐18057RostockGermany; ^6^ Department of Plant Science and Landscape ArchitectureDepartment of EntomologyUniversity of MarylandCollege ParkMD20742USA; ^7^ School of Natural SciencesZoology & Trinity Centre for Biodiversity ResearchTrinity College DublinDublin 2Ireland; ^8^ National Socio‐Environmental Synthesis Center1 Park PlaceAnnapolisMD21401USA; ^9^ Centre for Ecology and ConservationCollege of Life and Environmental SciencesUniversity of ExeterCornwall CampusExeterTR10 9FEUK; ^10^ Institute for Biodiversity and Ecosystem DynamicsUniversity of Amsterdam1090 GEAmsterdamThe Netherlands; ^11^ Biology Department MS‐34Woods Hole Oceanographic InstitutionWoods HoleMA02543‐1050USA; ^12^ Population Research InstituteDuke UniversityDurhamNC27708‐0309USA

**Keywords:** animal population ecology, comparative approach, matrix population model, open‐data, population growth rate (λ)

## Abstract

1. The open‐data scientific philosophy is being widely adopted and proving to promote considerable progress in ecology and evolution. Open‐data global data bases now exist on animal migration, species distribution, conservation status, etc. However, a gap exists for data on population dynamics spanning the rich diversity of the animal kingdom world‐wide. This information is fundamental to our understanding of the conditions that have shaped variation in animal life histories and their relationships with the environment, as well as the determinants of invasion and extinction.
2. Matrix population models (MPMs) are among the most widely used demographic tools by animal ecologists. MPMs project population dynamics based on the reproduction, survival and development of individuals in a population over their life cycle. The outputs from MPMs have direct biological interpretations, facilitating comparisons among animal species as different as *Caenorhabditis elegans*,* Loxodonta africana* and *Homo sapiens*.
3. Thousands of animal demographic records exist in the form of MPMs, but they are dispersed throughout the literature, rendering comparative analyses difficult. Here, we introduce the COMADRE Animal Matrix Database, an open‐data online repository, which in its version 1.0.0 contains data on 345 species world‐wide, from 402 studies with a total of 1625 population projection matrices. COMADRE also contains ancillary information (e.g. ecoregion, taxonomy, biogeography, etc.) that facilitates interpretation of the numerous demographic metrics that can be derived from its MPMs. We provide R code to some of these examples.
4. *Synthesis*: We introduce the COMADRE Animal Matrix Database, a resource for animal demography. Its open‐data nature, together with its ancillary information, will facilitate comparative analysis, as will the growing availability of databases focusing on other aspects of the rich animal diversity, and tools to query and combine them. Through future frequent updates of COMADRE, and its integration with other online resources, we encourage animal ecologists to tackle global ecological and evolutionary questions with unprecedented sample size.

## Introduction

Understanding the drivers and consequences of variation in reproduction and survival throughout the life cycle is fundamental for population biology, evolution, ecology and allied fields (e.g. Stearns 1989; Gaillard *et al*. 2005; Metcalf & Pavard 2007; Salguero‐Gómez & de Kroon 2010). Although demography is essential to understand and predict population dynamics, no single open‐data repository integrates this information for animal species world‐wide. This is mainly because most biological data sources are scattered and biological data types are heterogeneous (Hoffmann *et al*. 2014). Moreover, demographic data pose challenges for standardization due to the different formats (e.g. life table, matrix model, individual‐level records, population sizes, etc.) and terminology (Lebreton *et al*. 2012). This makes it challenging to create a single demographic data repository across multiple species. However, important efforts in this direction already exist, such as the Global Population Dynamics Database (GPDD, Inchausti & Halley 2001) or the Living Planet Index (LPI, Collen *et al*. 2009), BIDDABA (Lebreton *et al*. 2012) and the Primate Life History Database (PLHD, Strier *et al*. 2010) containing demographic information for birds and primates, respectively. These remarkable efforts are important contributions to population biology, but are limited in either demographic detail (GPDD, LPI) or taxonomic scope (BIDDABA, PLHD, WBI).

A mechanistic understanding of how and why populations invade, grow, decline, or go locally extinct, requires data and methods that provide insights into age‐/size‐/ontogeny‐based structure, such as Matrix Population Models (*MPMs* hereafter; Caswell 2001). MPMs have become a staple method describing the structured demography of animal populations. The widespread use of MPMs stems from their well‐understood mathematical foundations and tractability (Caswell 2001), coupled with the clear biological interpretations of the analytical outputs (e.g. growth rates, population structure and reproductive values; sensitivity and elasticity of demographic outputs; decomposition of treatment effects using LTRE analysis; measures of population viability and extinction risk; selection gradients in quantitative genetics and adaptive dynamics; and rates of spread of invasive species. See Caswell (2001), and Morris & Doak (2002) for detailed discussions and examples). Briefly, an MPM classifies the life cycle of a species into discrete stages and projects its population(s) through based on the probabilities of survival, transitions among stages and the contributions to sexual or clonal reproduction at each stage. The stages of the life cycle are typically chosen based on the biology of the species, and the projection interval can vary from days (e.g. Buston & García 2007) to years (e.g. Edmunds 2015), depending on the data available, species and questions.

**Figure 1:**
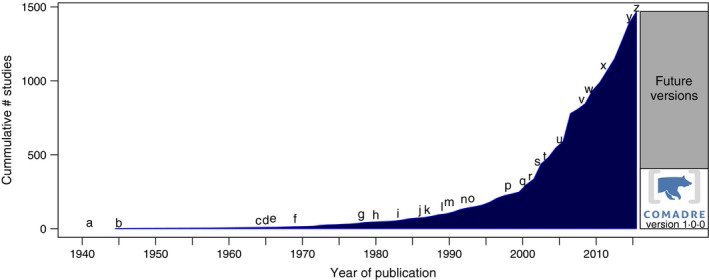
The cumulative number of studies published prior to 27.07.2015 containing animal matrix population models (MPMs). The right bar represents the total number of studies, including the number released in COMADRE version 1.0.0. Important events in the development of animal MPMs: the first (*a*,* b*) applications of matrix population models in demography (Bernardelli 1941; Leslie 1945), (*c*) and to humans (Keyfitz 1964), (*d*) introduction of theory for stage‐classified MPMs (Lefkovitch 1965), (*e*) first spatial MPM (Rogers 1966), (*f*) nonlinear, density‐dependent MPMs for animals (Pennycuick 1969; Rabinovich 1969), (*g*) sensitivity analysis for stage‐classified MPMs and calculation of selection gradients for animals (Caswell 1978), (*h*) bifurcation analysis of density‐dependent MPMs in animals (Levin & Goodyear 1980), (*i*) calculation of the stochastic growth rate from an animal MPM (Cohen, Christensen & Goodyear 1983), (*j*) formalization of elasticity analyses for MPMs (de Kroon *et al*. 1986), (*k*) application of elasticity analysis to conservation biology (Crouse, Crowder & Caswell 1987) and Life Table Response Experiment analysis (Levin 1987), (*l*) *Matrix Population Models: Construction, Analysis and Interpretation* edition 1 (Caswell 1989), (*m*) *Population Dynamics in Variable Environments* (Tuljapurkar 1990), (*n*) presentation of multistate mark–recapture methods for estimating stage‐structured MPMs in animals (Nichols *et al*. 1992), (*o*) development of MPM from photograph identification data (Brault & Caswell 1993), (*p*) an early study detailing uncertainty in MPMs (Caswell *et al*. 1998), (*q*) special feature on MPMs (Heppell, Pfister & de Kroon 2000), (*r*) *Matrix Population Models* 2nd edition (Caswell 2001), (s) publication of *Quantitative Conservation Biology: Theory and Practice of Population Viability Analysis* (Morris & Doak 2002) (*t*) first application of matrix integrodifference equations to examine animal invasion speeds (Caswell, Lensink & Neubert 2003), (*u*) first investigation of non‐equilibrium properties for MPMs (Caswell & Neubert 2005), (*v*) complete perturbation analysis for nonlinear animal MPMs (Caswell 2008), (*w*) introduction of individual stochasticity analyses for animal MPMs (Caswell 2009; Tuljapurkar, Steiner & Orzack 2009), (*x*) COMADRE established at the Max Planck Institute for Demographic Research, (*y*) COMPADRE Plant Population Database 3.0.0 released and (*z*) COMADRE Animal Matrix Database 1.0.0 released online in www.comadre-db.org.

**Figure 2:**
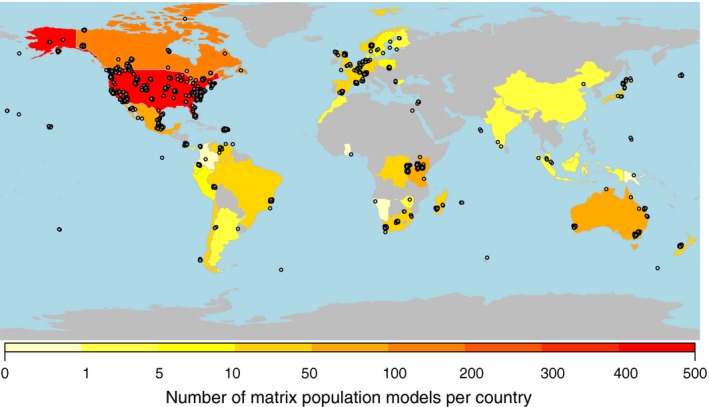
Geographic representation of animal demographic studies in COMADRE 1.0.0. The points represent study sites for which GPS coordinates are available; these have been jittered to highlight temporal replication within the same site, and close spatial overlap of certain studies. Countries with redder colour contain more matrix population models.

As is the case with plants (Salguero‐Gómez *et al*. 2015), a large number of MPMs have been published on species in the animal kingdom since the models were introduced in the 1940s (Bernardelli 1941; Leslie 1945) (Fig. 1). Underlining the general utility of MPMs, these models have been used to address diverse topics including conservation biology (e.g. Crouse, Crowder & Caswell 1987; Bessa‐Gomes *et al*. 2003; Colin & Lebreton 2005; Jenouvrier *et al*. 2012), evolutionary biology (e.g., Kawecki 1995; Gaillard *et al*. 2005; Gamelon *et al*. 2015), ecotoxicology (e.g., Charles *et al*. 2009), invasion biology (e.g., Neubert & Parker 2004) and resource management (e.g., Salomon *et al*. 2013). MPMs have been employed to study species as taxonomically distinct as *Caenorhabditis elegans*,* Loxodonta africana* and *Homo sapiens*, and in geographically diverse regions with studies in every major biome (Fig. 2a and b).

Despite the growing availability of published MPMs and the fact that such models are inherently comparable (Caswell 2001), there have been few attempts to use MPMs in comparative analyses. Notable exceptions are the work by Sæther & Bakke (2000) or Bessa‐Gomes *et al*. (2003) on birds, and Heppell, Caswell & Crowder (2000) and Gamelon *et al*. (2014, 2014) on mammals, Vélez‐Espino, Fox & McLaughlin (2006) on bony fish, Mollet & Cailliet (2002) on cartilaginous fish, and van de Kerk *et al*. (2013) on order Carnivora. These works illustrate the power of comparative approaches for robust generalizations by relating demographic estimates made from MPMs to interactions with the environment that form the basis for the evolution of life histories. One reason for the lack of comparative studies has historically been the paucity of readily available demographic data, compared with genetic data (e.g. Benson *et al*. 2013). This changed recently, when the COMPADRE Plant Matrix Database was released (Salguero‐Gómez *et al*. 2015). Since its foundation in 1990, COMPADRE has prompted over 35 comparative plant demography studies ranging from senescence (Silvertown, Franco & Perez‐Ishiwara 2001; Jones *et al*. 2014), to short‐term population dynamics (Stott, Townley & Hodgson 2011), to the link between functional traits and demography (Adler *et al*. 2014; Salguero‐Gómez *et al*. 2015). Here, we announce the release of COMPADRE's sister data base, the COMADRE Animal Matrix Database, containing MPMs and associated metadata from the animal kingdom.

The main objectives of the COMADRE team are (i) to find, digitize and systematically error‐check published animal MPMs and supplement them with additional information (Table 1); (ii) to offer such information on an open‐data framework; and (iii) to develop tools to facilitate comparative analyses. The data described here are available at www.comadre-db.org. In this paper, we briefly describe the COMADRE Animal Matrix Database and highlight the major differences and similarities with its sister data base, COMPADRE. In addition, we briefly report some geographic, taxonomic and modelling biases in the animal demography literature that are revealed by the compilation of MPMs in COMADRE. Finally, we share our vision for how this resource may expand and develop in the future, linking to other already existing open‐access data bases to address timely questions in animal ecology and evolution.

**Table 1:** Variables in the COMADRE Animal Matrix Database, organized by seven general aspects: taxonomy, source, details of study, geolocation and Matrix Population Model (MPM). Refer to Box 1 for the structural organization of the data in the R data object. A more detailed description can also be found in the user protocol of COMADRE at www.comadre-db.org. Variables 1‐27 contain study‐specific information and variables 28‐66 contain MPM‐specific information. Variables 1–54 are archived in comadre$metadata, variables 55–57 in comadre$matrixClass, variables 58–61 in comadre$mat and 62–67 in comadre$version in the COMADRE *R* data object (Box 1)

Aspect	Variable	Description
Taxonomy	1. *SpeciesAuthor*	Taxonomic species name as used by the author(s) in the publication. When more than one study exist for the same species, these are given sequential numeric suffixes (e.g. *Ursus_americanus, Ursus_americanus_2,* etc.)
	2. *SpeciesAccepted*	Currently accepted taxonomic name according to the Catalogue of Life (www.catalogueoflife.org). See the Supplementary Online Material S3 for an *R* script to check accepted and synonym names from *SpeciesAuthor* above
	3. *CommonName*	English common name of *SpeciesAccepted*
	4. *CoLCheckOK*	Whether the taxonomy detailed here has been verified at the Catalogue of Life
	5. *CoLCheckDate*	The date (*DDMMYYYY*) that the taxonomy was checked at the Catalogue of Life
	6. *Infraspecific*	Taxonomic infraspecific name of *SpeciesAccepted*, as used by the author
	7. *SpeciesEpithetAccepted*	Taxonomic species epithet of study species, as per Catalogue of Life
	8. *GenusAccepted*	Taxonomic genus of study species, as per Catalogue of Life
	9. *GenusAuthor*	Taxonomic genus of study species, as in *SpeciesAuthor*
	10. *Family*	Taxonomic family of study species
	11. *Order*	Taxonomic order of study species
	12. *Class*	Taxonomic class of study species
	13. *Phylum*	Taxonomic phylum of study species
	14. *Kingdom*	Taxonomic kingdom of species
Source of information	15. *Authors*	Last names of full authorship in study
	16. *Journal*	Abbreviated journal of publication (www.abbreviations.com/jas.php), otherwise stated as ‘PhD thesis’, ‘MSc thesis’, ‘BSc thesis’, ‘Book’, ‘Report’ or ‘Internet’
	17. *YearPublication*	Year of publication of source
	18. *DOI/ISBN*	Digital object identifier (for manuscripts) or international standard book number (for books), when available; old publications do not have an assigned DOI. An *R* script is also provided to obtain full citation from manuscripts based on DOI (See Online Supplementary Materials 3)
	19. *AdditionalSource*	If additional information was obtained from a secondary source, the abbreviated citation is included here (First author's first last name, abbreviated journal name and publication year; e.g.: ‘Naujokaitis‐Lewis *Cons Biol* 2009’ for *Canis latrans)*
Details of the study	20. *StudyDuration*	Years of observation of the population dynamics of the species, calculated as *StudyEnd* – *StudyStart* + 1 (e.g. 2005–2000 + 1 = 6). This does not mean the data were collected annually – see *AnnualPeriodicity* below
	21. *StudyStart*	Year the study started
	22. *StudyEnd*	Year the study ended
	23. *AnnualPeriodicity*	Frequency with which seasonal or annual MPMs were constructed (e.g. 1: once per year; 2: twice per year; 0·2: once every five years)
	24. *NumberPopulations*	Number of populations examined in the study – These may not match the number of populations with MPMs in COMADRE 1.0.0 if the author has not made available all of the MPMs
	25. *MatrixCriteriaSize*	Whether and on which biometric aspects of the species was the MPM constructed (e.g. height)
	26. *MatrixCriteriaOntogeny*	Whether some aspect of developmental stage of the species was used to construct the MPM (e.g. juvenile, reproductive adult)
	27. *MatrixCriteriaAge*	Whether some aspect of developmental stage of the species was used to construct the MPM (e.g. 0, 1, 2 years old)
Location	28. *MatrixPopulation*	Name(s) of populations from which the MPM was constructed. When no population name is provided in the source, the name of closest geographic landmark or letters in alphabetical (e.g. ‘A’, ‘B’, ‘C’…) or numerical order (e.g. ‘1’, ‘2’, ‘3’…) are used
	GPS Location	
	29. *LatDeg*	Latitudinal degrees of study population
	30. *LatMin*	Latitudinal minutes of study population
	31. *LatSec*	Latitudinal seconds of study population
	32. *LatNS*	Latitudinal cardinal direction: North or South
	33. *LonDeg*	Longitudinal degrees of study population
	34. *LonMin*	Longitudinal minutes of study population
	35. *LonSec*	Longitudinal seconds of study population
	36. *LonWE*	Longitudinal cardinal direction: West or East
	37. *Altitude*	Altitude of study population (in metres) obtained from Google Earth
	38. *Country*	Country or countries where the study population was studied. Only countries currently accepted by the United Nations according to the ISO 3 list were used (http://unstats.un.org/unsd/tradekb/Knowledgebase/Country-Code)
	39. *Continent*	Continent of the study population
	40. *Ecoregion*	Description of the terrestrial or aquatic ecoregion, corresponding to Olson *et al*.'s classification (2001), where the study took place. When the study is undertaken in its majority under controlled, indoor conditions (e.g. laboratory, glasshouse), this is noted as ‘*LAB*’
Details of matrix population Model	41. *StudiedSex*	Sex(es) considered to construct the MPM (Fig. 3b)
	42. *MatrixComposite*	MPMs were differentiated between matrices that correspond to a given single population, single treatment and single annual period (‘Individual’; Fig. 3a), to a single population, treatment and intraannual period (‘Seasonal’), to a MPM that is the result of element‐by‐element arithmetic mean (‘Mean’), or where the individual‐level data were pooled to construct a MPM over various periods, populations and/or treatments (‘Pooled’). We must note that by default we calculated the mean MPM when all individual MPMs in the study were made available. The pooled and mean matrices for all the individual, unmanipulated (see *MatrixTreatment*) MPMs coincide when the sample sizes and stage distributions at time *t* are the same across all the individual MPMs. Mean MPMs were only calculated by us for unmanipulated individual matrices below
	43. *MatrixTreatment*	Treatment under which the demographic data used to parameterize the specific MPM was exerted. We specified ‘*Unmanipulated*’ as those matrices where no human‐led experimentation was carried out (Fig. 3f). Users are encouraged to carefully examine variable *MatrixObservation* (below) for additional pertinent information
	44. *Captivity*	Whether the study species was in its wild setting, or under control conditions (e.g. glasshouse, botanical garden) for most of the demographic data that were collected (Fig. 3e)
	*Start and end of study period*	
	44. *MatrixStartYear*	Beginning year *t* for MPM ***A*** describing the population dynamics between time *t* and year *t + 1*
	45. *MatrixStartSeason*	Beginning season *s* for seasonal MPM ***B*** describing the population dynamics between season *s* and season *s + 1*
	46. *MatrixStartMonth*	Beginning month *m* for seasonal MPM ***B*** describing the population dynamics between month *m* and month *m + 1*
	47. *MatrixEndYear*	End year *t + 1* for MPM ***A*** describing the population dynamics between time *t* and time *t + 1*
	48. *MatrixEndSeason*	End season *s + 1* for seasonal MPM ***B*** describing the population dynamics between seasons *s* and season *s + 1*
	49. *MatrixEndMonth*	End month *m + 1* for seasonal MPM ***B*** describing the population dynamics between month *m* and month *m + 1*
	50. *MatrixSplit*	To facilitate the calculation of various demographic properties (e.g. life expectancy η_*e*_, mean age at first reproduction *L*_α_, vital rate sensitivities, etc.), the MPM ***A*** (*matA*, below) has been split into survival (*matU*), sexual (mat*F*), and clonal reproduction (*matC)* submatrices when sufficient information was provided in the source. In 2·9% of the cases, insufficient information led to us not been able to split ***A*** into ***U***,***F*** and ***C***. This matrix is referred to as *Indivisible* (Fig. 3c)
	51. *MatrixFec*	In some instances, the sexual reproductive component of the life cycle of the organism (see *matF* below) is not modelled either because it is not of interest to the researcher or because it was unfeasible
	52. *Observation*	Relevant observation that the user should have in mind when analysing and interpreting the MPMs. In the present version, >50% of the matrices made available in this version have observations. Observations include, for instance, warnings about the description by the author of an ‘*Unmanipulated*’ population that some researchers may wish to treat as a treatment (e.g. natural fires), among others
	53. *MatrixDimension*	Dimension of the MPM
	54. *SurvivalIssue*	Reports maximum stage‐specific survival in the submatrix ***U*** (below). If this value > 1, users are encouraged to carefully evaluate the matrix
	55. *MatrixClassAuthor*	Classification of the stages in the life cycle of the study species as described by the author
	56. *MatrixClassOrganized*	Standardization of *MatrixClassAuthor* into three stages: *prop* for propagules, *dorm* for dormant individuals, and *active* for individuals active, established individuals. We standardized *MatrixClassAuthor* in this way to facilitate cross comparisons of various general life cycle stages. Note that further general classifications are possible, for instance, distinguishing reproductive individuals from non‐reproductive individuals by evaluating the ***F*** and ***C*** submatrices
	57. *MatrixClassNumber*	Sequence of numbered classes from 1 to *MatrixDimension*
Matrix Population Model	58. *matA*	MPM including demographic processes that depend on survival (*SubMatrixU* below), sexual reproduction (if pertinent and available; *SubMatrixF* below), and clonal reproduction (if pertinent and available; *SubMatrixC* below; Fig. 3)
	59. *matU*	Submatrix population model describing only survival‐dependent demographic processes (e.g. seedbank, stasis, progression, retrogression, vegetative dormancy, etc.). Matrix elements corresponding to sexual and clonal reproduction are filled with zeros
	60. *matF*	Submatrix population model describing only sexual reproduction. All other matrix elements are filled with zeros
	61. *matC*	Submatrix population model describing only clonal reproduction. All other matrix elements are filled with zeros
Version	62. *Version*	Version of COMADRE. Currently 1.0.0
	63. *DateCreated*	Date of compilation of version. Currently October 2nd 2015
	64. *NumberSpeciesAccepted*	Total number of species taxonomically accepted in COMADRE. Currently 345 species
	65. *NumberStudies*	Total number of studies in COMADRE. Currently 402 studies
	66. *NumberMatrices*	Total number of MPMs in COMADRE. Currently 1625 MPMs
	67. Agreement	Link to the user agreement of the data base

## Animal matrix population models: a historical perspective

The accumulated number of publications reporting MPMs for animals has increased dramatically since MPMs were introduced in the 1940s (Fig. 1). Important contributions to this history come from the introduction of new *types* of MPMs, and new *methods* for analysing them.

Matrix population models were largely ignored for twenty years after the work of Leslie (1945) (but see for instance Thompson 1959). The rediscovery of MPMs in the 1960s can be credited to Keyfitz (1964), Lefkovitch (1965) and Rogers (1966), whose works focused on animals. Keyfitz (1964) presented MPMs as tools for projecting population growth; his book (Keyfitz 1968) influenced a generation of animal ecologists. The first presentations of MPMs had assumed that age was the only individual state (*i*‐state; Metz & Diekmann 1986) variable. Lefkovitch (1965), based on studies of laboratory populations of stored product insect pests, explicitly proposed stage‐classified models based on the life cycle stages of insects. Rogers (1966) introduced spatial, or multiregional, models for human populations, classifying individuals by age and spatial location, and modelling survival, fertility and migration.

Other types of MPMs were introduced in the following years. The first seasonal, periodic MPM appeared in 1964 (Darwin & Williams 1964) in a study of seasonal harvesting as a control strategy for rabbits. The first density‐dependent models appeared in 1969: Pennycuick (1969) analysed a population of great tits (*Parus major*), while Rabinovich (1969) compared several density‐dependent models, including a MPM to analyse laboratory populations of a parasitoid wasp. The first stochastic model for an animal population was the analysis by Cohen, Christensen & Goodyear (1983) on recruitment fluctuations in striped bass (*Morone saxatilis*). Invasion models, using matrix integrodifference equations, were first applied to bird populations by Caswell, Lensink & Neubert (2003).

Analytical methods have developed in parallel with their applications to animal populations. Some of these developments have provided new ways of constructing models (photograph‐identification methods, mark–recapture methods, vec‐permutation matrix methods). Others have provided ways to extract additional information from the resulting MPM (perturbation analyses; LTRE decompositions; stability and bifurcation for nonlinear models; Markov chain methods for the analysis of longevity, heterogeneity and individual stochasticity; reactivity and amplification analyses). Further important contributions are detailed in Fig. 1.

The COMADRE Animal Matrix Database was founded at the Max Planck Institute for Demographic Research (MPIDR) (Appendix S1, Supporting information). The motivation for the creation of a data base containing MPMs for animals was based on substantial contributions of its sister data base, the COMPADRE Plant Matrix Database (Salguero‐Gómez *et al*. 2015), to plant ecology and evolution. Four years after its foundation, the COMADRE digitization team has digitized, standardized, error‐checked and supplemented information contained in over 400 species. As with the commitment for its plant sister data base, more data will be released periodically (Fig. 1) at www.comadre-db.org.

## What is in the COMADRE portal?

The COMADRE portal (www.comadre-db.org) provides access to the data to important news (e.g. version releases), the user's guide and digitalization protocol, announcements on workshops and open‐access scripts for analyses. The current version of the COMADRE data is provided in a structured *Rdata* object format (Box 1, Fig. S1); this will migrate to an SQL infrastructure eventually. The information in COMADRE is obtained mostly (>99%) from published peer‐reviewed manuscripts obtained from searches of ISI, Scopus and Google Scholar with keywords frequently used in publications containing MPMs (e.g. ‘*elasticity*’, ‘*sensitivity*’, ‘*LTRE*’, ‘*population growth rate*’, ‘*matrix population model*’, ‘*projection matrix*’). The portal contains a list of all species included in the current release of the data, as well as those still being digitized/error‐checked. Users are encouraged to email their works containing MPMs at comadre-contact@demogr.mpg.de if not cited in the aforementioned list. The demographic information is digitized, re‐organized (see equation 2) and error‐checked (below), and then supplemented with additional sources (e.g. taxonomy, ecoregion; Table 1). The COMADRE user's guide details the organization of the data object, the meaning and range of possible values for these variables, as well as information on error‐checks and quality controls that are carried out. Additionally, Frequently Asked Questions (FAQs) can be found at the online portal (http://www.comadre-db.org/Help).

The basic data item of COMADRE is the population projection matrix. A basic (*i.e*. linear and time‐invariant) MPM can be written as

(eqn 1) n(t+1)=An(t)

where ***n*** is a vector describing the abundance of a set of age/size/ontogenetic classes and ***A*** is a population projection matrix. The structure of the projection matrix ***A*** depends on the choice of life cycle stages and the projection interval.

In COMADRE, the projection matrix is decomposed as

(eqn 2) A=U+F+C

where ***U*** is the matrix describing transitions and survival of extant individuals, and ***F*** and ***C*** are the matrices describing production of new individuals by sexual and clonal reproduction, respectively. Some studies do not measure reproduction, reporting only the transition matrix ***U***. In these cases, this is reflected in the variable *MatrixFec* (see Table 1 and COMADRE User's Guide for details). The column sums of ***U*** give the survival probabilities of the stages, and thus should not exceed 1 (below).

The simple model (eqn 1) can be extended in several ways. *Seasonal* MPMs divide the year into seasons and report a projection matrix ***B**(i)* for season *i* during the *m* seasons/periods in the year; the data base entries for such seasonal models report all of the seasonal matrices, and when necessary, we also calculated the consequent annual matrix for inclusion in COMADRE:

(eqn 3) A=B(m)B(m−1)…B(2)B(1)

*Stochastic, density‐dependent and environment‐dependent* MPMs are increasingly common in animal studies (e.g. Cushing *et al*. 2003; Jenouvrier *et al*. 2009; Hunter *et al*. 2010; Barraquand *et al*. 2014). In such cases, the MPM can be written as in equation 4,

(eqn 4) n(t+1)=A[t,n(t),E(t)]n(t)

where ***E***(*t*) corresponds to environmental conditions. Such a model is associated not with a single projection matrix, but with a function that returns an ***A*** matrix given a time and/or environment and/or population vector. Because such functions require a different data structure, such models are not directly included in COMADRE 1.0.0. However, in some cases, static MPMs are presented at particular values of density or environmental conditions, as specified in the variable *Treatment* (Table 1). These are not stochastic, density‐dependent or environment‐dependent models, although they might eventually be used to construct such models.

Associated with each MPM is a set of descriptive information (metadata). These metadata are contained in the *R* object *COMADRE_v.1.0.0.Rdata* as a list object that contains four further subhierarchical objects: *metadata*, *matrixClass*, *mat* and *version* (Box 1; See User's guide). The *metadata* object (a data.frame) can be accessed in *R* with the command comadre$metadata, and it includes information about taxonomy, additional details of the study including its source, geolocation and some details about the specific MPMs (Variables 1 through 54 inTable 1). Information about the classes used to construct the specific MPM is contained in the matrixClass object (a list of data.frames), which can be accessed with the *R* command comadre$matrixClass. The MPMs are held in a list of lists that can be retrieved with the command comadre$mat. Data pertaining to particular MPMs can be obtained using R’s standard data indexing facilities; for instance, comadre metadata[n,] and comadre$matrixClass[[n]] will return the metadata and class information pertaining to the *n*th matrix (comadre$mat[[n]]). This is illustrated in Box 1, where we demonstrate how to obtain data for the snowshoe hare (*Lepus americanus*; Meslow & Keith 1968). We provide further examples on how to query COMADRE to run comparative analyses in Appendix S4.1–4.7. The last object within COMADRE is the list version, which contains summary information about the version for replicability purposes, as well as a link to the User's Agreement.

In some cases, the original data source provided information that allowed us to split the full life cycle matrix (***A***) into non‐reproductive, survival‐dependent transition probability processes (***U***), sexual reproduction (***F***), and clonal reproduction, (***C***) as described in equation 2. Whether this was possible or not is indicated in by the variable *MatrixSplit* (Table 1). This set of four matrices (***A***,***U***,***F*** and ***C***) are stored as a list (with elements named matA, matU, matF and matC, respectively) within the elements of the comadre$matlist. Thus, the matrices can be obtained with ease: comadre mat[[n]] matA, comadre$mat[[n]]$matU, etc. Splitting the matrices in this way allows for automated calculation of demographic output of various kinds from hundreds of records in a few seconds (Appendix S4).

## COMADRE and COMPADRE: similarities and differences

The core data in both COMADRE and COMPADRE are the projection matrices that appear in MPMs. A comparison of Table 1 in this manuscript and Table 1 in the introduction to COMPADRE Plant Matrix Database (Salguero‐Gómez *et al*. 2015) reveals a number of similarities. Moreover, the data quality controls are the same for COMPADRE and for COMADRE (below).

Despite the similarities, biological differences between plants and animals mean that animal data cannot be fully accommodated by the data base framework of COMPADRE. The key differences between the two data bases are as follows: (i) the variables *GrowthType*,* DicotMonocot* and *AngioGymno* are specific to plants and so do not exist in COMADRE; (ii) variables related to taxonomic validation and cross‐referencing (1–14 in Table 1) were crosschecked with The Catalogue of Life (CoL) (http://www.catalogueoflife.org) in COMADRE, instead of The Plant List (http://www.theplantlist.org) as done for COMPADRE; and (iii) we have added in COMADRE the variable *MatrixFec*, which indicates whether the reproductive component (matrices ***F*** and/or ***C***) of the matrix model is missing or not. This variable was not present in COMPADRE 3.0.0, but it is now in 3.2.1; (iv) unlike in COMPADRE, where we reconstructed a phylogeny for plant species, a tree for most animal species in COMADRE has been recently published (Hedges *et al*. 2015). Furthermore, species‐level resolved trees also exist for some taxonomic groups such as mammals (Bininda‐Emonds *et al*. 2007), birds (Jetz *et al*. 2012) or reptiles (Pyron, Burbrink & Wiens 2013).

## Error‐checking and cautionary notes

To facilitate transparency in the data entry and error‐checking process, we provide the protocol used internally by our team in the Online Appendix (Supporting information). Most of these checks were detailed in the publication of the COMPADRE Plant Matrix Database (Salguero‐Gómez *et al*. 2015). Important error‐checks include making sure that equation 2 remains correct for all MPMs and that stage‐specific survival (*i.e*., the column sums of the matrix ***U***) does not exceed 1. In order to eliminate potential typographical errors, a double‐blind check, whereby the same MPM is digitized by two team members, each unaware that the other is doing so, and of what the results are, is carried out for *ca*. 50% of the original MPM sources. Additionally, each datum digitized by the COMADRE team is reviewed and error‐checked three times by the team leaders before the data are released. We do note that typos may remain despite our efforts. Users are encouraged to contact us at comadre-contact@demogr.mpg.de to inform us of potential errors.

Users wishing to run ‘big data analyses’ with COMADRE must keep in mind that, although hundreds of open‐data demographic records are available in COMADRE, these may not all be appropriate for a particular analyses. Data selection is perhaps the most important step in comparative research; thus, users must ask carefully which variables in Table 1 are irrelevant, relevant or missing for their research goals. Careful data selection criteria will allow for *fair* large analyses, the gold standard in comparative analyses. For instance, does the research require demographic data from various populations per species or is one population per species sufficient (*NumberPopulations*)?.Should there be a minimum threshold to the study length (*StudyDuration*)? We provide several examples of R code in the Online Appendix 4 to help users subset data based on various selection criteria.

We emphasize the importance of variables *SurvivalIssue* and *MatrixFec* (Table 1) to facilitate detection of issues related to survival‐ or reproduction‐dependent life history traits, respectively. *SurvivalIssue* reports the maximum stage‐specific survival value in the ***U*** matrix. This is important because the stage‐specific survival of any column sums of the ***U*** matrix is constrained to be between 0 and 1, and values greater than 1 render most analyses impossible, particularly those on survival and longevity. During data entry, when probabilities exceeded the error margin for rounding error and were considerably greater than 1, contributing authors were contacted for clarification. In some cases (<13% of MPMs with this issue), these personal communications have resulted in amendments from the originally published matrices or in the re‐assignment of proportions of each matrix element in **A** to the submatrices ***U***,***F*** and ***C*** (eqn 2). MPMs where this issue is still pending (*IssueSurvival* >1) may contain information provided by the authors in the variable ‘Observation’ (Table 1). Currently, only 0·9% of the MPMs (15 of the 1625) in version 1.0.0 have at least one stage‐specific survival >1. *MatrixFec* = ‘No’ is used to indicate MPMs for which reproduction was not modelled, to distinguish those cases from instances where reproduction was measured, but no recruitment occurred in *t *+* *1. In the former case, the MPM cannot be used to quantify metrics that depend on reproduction, such as population growth rate λ, its elasticities/sensitivities, damping ratio ρ and net reproductive rate. However, other metrics are still valid in these models (e.g. life expectancy η_*e*_ from *matU*; Table 1). In version 1.0.0, only 4·6% of the MPMs (75 of 1625) contain this issue in the submatrix ***F***.

The study designs, data sources and estimation procedures used to estimate MPMs are incredibly diverse. The research questions that can be asked and the analytical methods that exist to address them are equally diverse. Not all of the information relevant to a given analysis can be included in COMADRE. Just as with any scientific study, a user must take care that the data used are appropriate for the question and the analysis. This may require revisiting original sources (Appendix S5) to clarify aspects of the original study. A non‐exhaustive list of potential issues includes the effect of processes not included in the model (e.g. permanent emigration that may be confounded with mortality), the relative precision of estimates obtained by different methods (e.g. the difference between sessile and motile organisms) and the methods used to obtain measurements of age or stage. Reproduction in MPMs in COMADRE has not been categorized into pre‐breeding, post‐breeding or birth‐flow categories (Caswell 2001, p. 130); these classifications are not relevant to all studies, but may be of interest for some purposes. Thus, although MPMs from peer‐reviewed publications are included only after passing our error‐checks, some may still contain issues related to the original author's calculations.

## Scope and coverage of COMADRE

COMADRE contains an unprecedented sample of information on animal population dynamics: 1625 MPMs from 402 studies corresponding to 345 taxonomically accepted species according to the Catalogue of Life (Appendix 4.2). This represents a substantial improvement in sample size and ancillary information to date (Table 1), including important comparative works examining various aspects of life history traits and population dynamics of mammals (50 species in Heppell, Caswell & Crowder 2000; 111 in Gamelon *et al*. 2014), birds (49 species in Sæther & Bakke 2000), fish (88 species in Vélez‐Espino, Fox & McLaughlin 2006) and order Carnivora (285 species in van de Kerk *et al*. 2013). It must also be noted that the information analysed in the aforementioned studies was not made open access with the exception of van de Kerk *et al*. (2013), who archived it into COMADRE.

COMADRE offers a broad geographic coverage of animal population dynamics (Fig. 2). Information in COMADRE 1.0.0 includes MPMs from all continents except Antarctica – although MPMs for Antarctic species do exist and will be released in future version of COMADRE (e.g. Emperor penguin, Jenouvrier *et al*. 2012; Antarctic petrel, Decamps *et al*. 2015). Importantly, geographic gaps do exist in our knowledge of animal demography in certain regions, including Oceania (8·11% of MPMs), and Asia (2·49%; Fig. 2b). Together, the USA (31·7%), Canada (8·7%), Australia (5·3%) and Kenya (4·8%) comprise over half the MPMs in COMADRE 1.0.0. Unfortunately, few studies report MPMs from biodiversity hotspots such as Honduras, Guatemala, the Democratic Republic of Congo, Paraguay, India and Indonesia. Furthermore, some developed countries, such as Saudi Arabia, Italy, Greece, Ireland and Brazil, are under‐represented.

**Figure 3:**
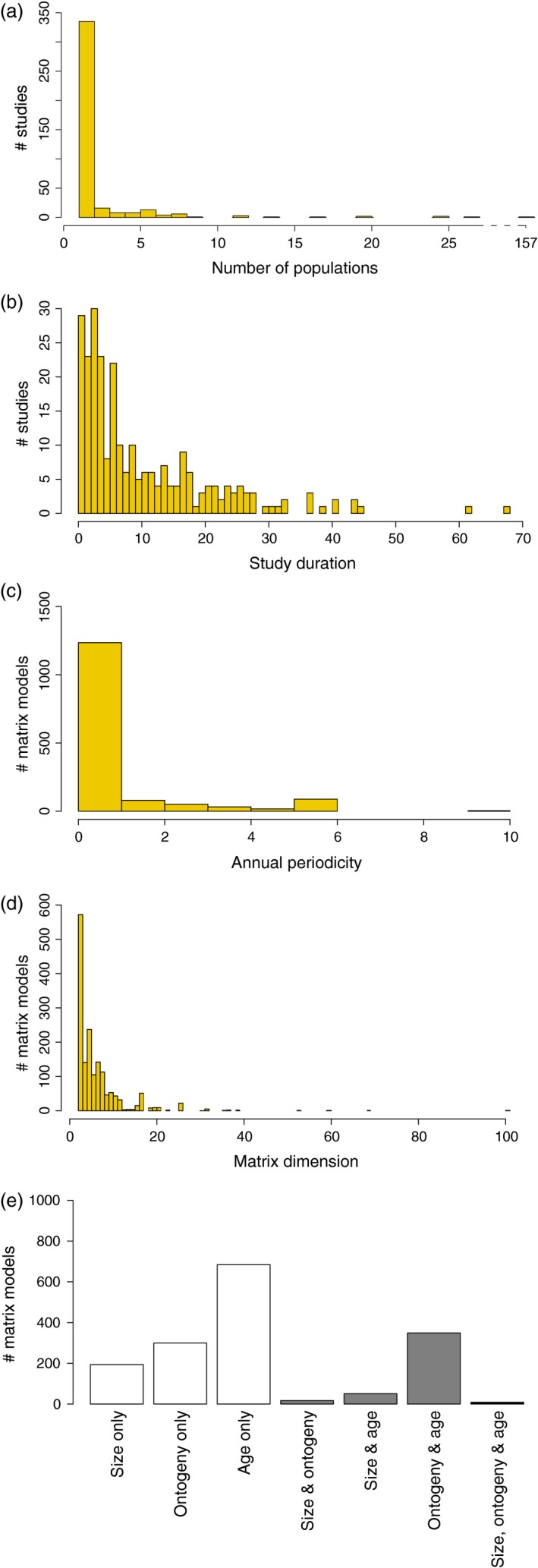
Most of the studies in COMADRE examine only one population per species (a; see *NumberPopulations* in Table 1), although they can achieve remarkable durations (in years) (b; *StudyDuration*). Most of the matrices in the data base detail annual population dynamics (c; *Periodicity*), with few (2–6) life cycle stages (d; *MatrixPeriodicity*) and these tend to be based on age and or ontogeny (e; *MatrixCriteriaSize, MatrixCriteriaOntogeny* & *MatrixCriteriaAge*). Panel A's *x*‐axis is broken between 27 and 156 populations/study.

Individual and seasonal population projection matrices (Table 1 #42) together total over 50% of the matrices in COMADRE 1.0.0, representing unique combinations of studies × species × populations × treatments × periods (Fig. S2A). The remaining 772 projection matrices are element‐by‐element arithmetic means of other matrices, or constructed based on data from multiple sources (‘pooled’). Given the spatial (Fig. 3a), interannual (Fig. 3b) and intraannual replication (Fig. 3c) in many studies, the high proportion of mean and pooled matrices suggests a tendency in animal demographic studies to publish only summary MPMs. We encourage authors to publish their original MPMs in the supplementary materials for their papers. Authors can archive MPMs in COMADRE by submitting them directly at comadre-contact@demogr.mpg.de.

Most studies in COMADRE are of natural populations in the wild (89%, Fig. S2B) and under unmanipulated conditions (i.e. no researcher‐imposed treatment) (80%; Fig. S2C). Most of the demographic studies in COMADRE are based on females only (67%; Fig. S2D); this is common practice in animal demographic studies (particularly in mammals), as quantifying reproduction and assigning paternity are challenging in males. We have noted, in the variable ‘Observations’ (Table 1 #52), when the primary sex ratio was stated by the author as differing from 1:1 (female:male). For the vast majority of matrices (95%; Fig. S2E), we have successfully split the full matrix ***A*** into its subcomponents of survival (***U***), sexual reproduction (***F***) and clonal reproduction (***C***) as per equation 2, and only 4·6% of the MPMs do not incorporate reproductive information (Fig. S2F).

The data in COMADRE 1.0.0 represent a wide range of animal groups (Fig. S2G). However, even though the digitization in COMADRE has not been prioritized by taxonomic group or geographic region, some strong taxonomic biases exist. Mammals represent 44·8% of the MPMs in the current version of COMADRE, followed by birds (17·5%), bony fish (Actinopterygii 10·6%) and reptiles (6·1%). COMADRE includes few MPMs for amphibians (0·9%), despite global concerns for their conservation status (Beebee & Griffiths 2005; Wake & Vredenburg 2008) or for insects (2·7%), despite their high species richness, estimated to comprise the majority of the animal kingdom (Hedges *et al*. 2015). The latter is particularly surprising a important early developments of MPMs focused on insects thanks to their clearly structured population dynamics (Lefkovitch 1965; Rabinovich 1969). This may reflect the widespread use of seasonal life table methods for insects, due to their annual life cycles (e.g. Dempster 1975), prior to the introduction of periodic matrix models specifically aimed at annual organisms (Caswell 2001; Chap. 13.2). Aside from bony fish, we lack significant amounts of demographic information on marine organisms in COMADRE, including corals (5·7%), bivalves (1·3%), sponges (0·8%), sea urchins (0·1%) and cartilaginous fish (0·2%). No information in COMADRE 1.0.0 exists for the order Struthioniformes (kiwis, emu, ostriches, etc.) or infraclass Marsupialia (kangaroos, wallabies, possums, opossums, wombats, etc., with the exception of the koala *Phascolarctos cinereus;* Baxter *et al*. 2006; Ng *et al*. 2014; Appendix S4.1).

The replication of studies through time and over space is highly variable in COMADRE. Yet, the average duration of studies in COMADRE 1.0.0 (10·97 years ± 0·68 SE; Fig. 3b, Fig. S3) is greater than in plant MPM studies (5·60 ± 0·23 years; Salguero‐Gómez *et al*. 2015). Long duration is essential for many demographic studies in the animal kingdom, as some animals, such as the clam *Arctica islandica*, giant tortoises (*Geochelone nigra, G. gigantea*), some rockfish species (e.g. *Sebastes aleutianus*) and the bowhead whale (*Balaena mysticetus*), can reach over 150 years of age (de Magalhaes & Costa 2009). Notable demographic studies using MPMs parameterized with long time series include *Vipera aspis* (17 years, Altwegg *et al*. 2005), *Ursus americanus* (22 years, Mitchell *et al*. 2009), *Delphinus delphis* (35 years, Mannocci *et al*. 2012), *Recurvirostra avosetta* (40 years, Hill 1988), *Elatobium abietinum* (41 years, Estay *et al*. 2012), *Marmota flaviventris* (44 years, Ozgul *et al*. 2009), *Haliaeetus albicilla* (62 years, Krüger, Grünkorn & Struwe‐Juhl 2010), *Diomedea exulans* (51 years, Barbraud *et al*. 2013) and *Aythya affinis* (72 years, Koons *et al*. 2006).

In contrast to the duration, the average number of populations considered in each study is relatively low, averaging 2·52 ± 0·42 (SE). The low spatial replication currently limits much‐needed understanding of the geographic variability of demographic rates within species. It is perhaps not surprising that the animal studies with highest spatial replication in COMADRE 1.0.0 focus on humans, with the foundational archive of human MPMs compiled by Keyfitz & Flieger (1968), which covers populations from 157 countries. We note that analyses of spatial and other kinds of variability in animal population studies are becoming more sophisticated due to the use of model selection methods to explicitly include environmental variables (e.g. Thomson, Cooch & Conroy 2009), and the concept of ‘spatial replication’ used in plant studies may acquire a different meaning to that used in most animal studies.

## Unlocking global analyses

I'm not interested in your data; I'm interested in merging your data with other data. Your data will never be as exciting as what I can merge it with

Tim Berners‐Lee

The potential of the COMADRE Animal Matrix Database does not reside exclusively in its hundreds of MPMs, or on the frequent future updates with new species and studies, but also in the many outputs that can be derived from them, and the possibility to place them in a broader spatial, ecological and evolutionary context using other open‐data sources. Users of COMADRE can find *R* scripts to manipulate and interact with MPMs and derive basic demographic outputs (Appendix S4 and updated scripts in our GitHub repository). Users are encouraged to explore these and other open‐source libraries (Stubben & Milligan 2007; Stott, Hodgson & Townley 2012; Metcalf *et al*. 2013), and to apply the rich repertoire of analytical methods for MPMs (Caswell 2001; Morris & Doak 2002).

The schedules of growth, survival and reproduction and the associated population performance metrics available through COMADRE will enable further comparative analyses of life history variation and population performance relative to the environment. For example, information in COMADRE could be integrated with existing repositories for other data such as genetic sequences (GenBank; Benson *et al*. 2013), distribution and occurrences (GBIF; Flemons *et al*. 2007), and conservation status and threats (BirdLife, http://www.birdlife.org/datazone; IUCN Red List, http://www.iucnredlist.org). Data on species‐level life history traits are also available for specific taxonomic groups including vertebrates (AnAge; de Magalhaes & Costa 2009), mammals (Ernest 2003; PanTHERIA, Jones *et al*. 2009), amphibians (Trochet *et al*. 2014), fish (FishBase) and reptiles (SCALETOOL, www.scale-project.net). In addition to the rapidly growing body of data, a diverse set of tools are emerging that will facilitate these large‐scale comparative analyses including the *R* packages *taxize* (Chamberlain & Szöcs 2013), *letsR* (Vilela & Villalobos 2015) which facilitate taxonomic matching and macroecological analyses, respectively.

We hope that the compilation of demographic data in COMADRE will also enable ecologists to identify the gaps in our knowledge in animal population dynamics and will catalyse new studies at broad spatial scales. The open‐data nature of both COMPADRE and COMADRE will facilitate further comparative demographic analyses across plant and animal kingdoms (see Jones *et al*. 2014) enabling tests of life history and population dynamics theory across a wide range of species with contrasting life histories, mobility/dispersal and architecture. We suggest that researchers revisit the canonical tenets of animal life history to confront established theories with data compilations that are vastly richer than was available 30 years ago. We have already identified over 900 additional animal species with MPMs (Fig. 1), and our ongoing efforts will release them as they become fully digitized, error‐checked and supplemented in the coming years. Finally, researchers using the data archived in COMADRE are strongly encouraged to cite also the original sources (Appendix S5).

Box 1 **The COMADRE Animal Matrix Database is organized into four main branches: (i) metadata, a data. frame that contains information pertinent to the species and study of each matrix population model (MPM), (ii) mat, a list containing the MPMs, (iii) matrixClass, a list with the descriptors of the stages used to describe the life cycle from which each MPM resulted, and (iv) version, a list with metadata about the version of COMADRE (See Table 1). Example outputs are presented below in each branch. See Figure S.1 for a full depiction of all variables in Table 1 for a given search. Photo credit: NPS Photo Tim Rains.**

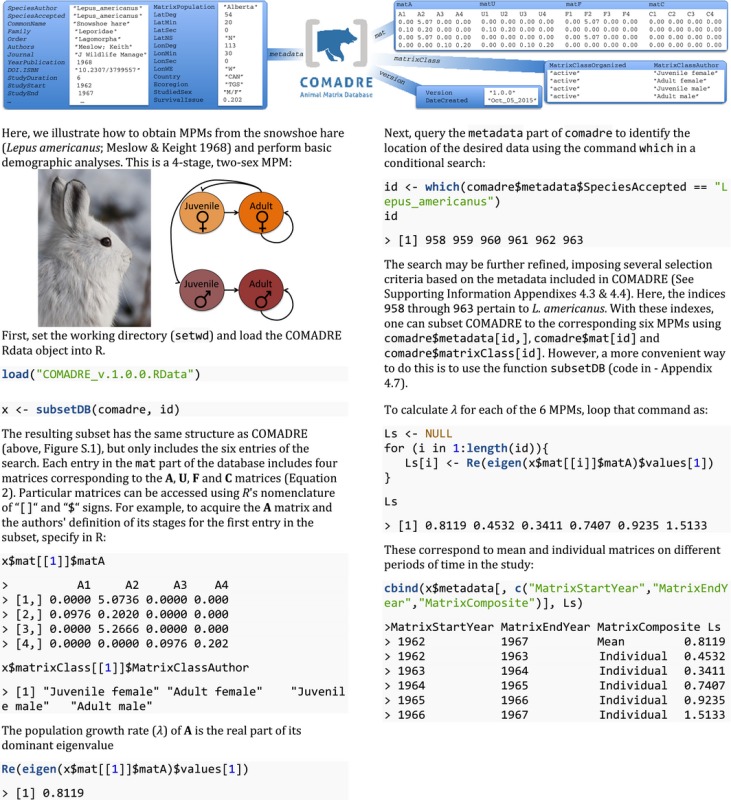

## Data accessibility

The data associated with this manuscript can be accessed at www.comadre-db.org.

## Supporting information


**Appendix S1.** Supporting Online Figures.
**Appendix S2.** Constituents of COMADRE.
**Appendix S3.** COMADRE user's guide.
**Appendix S4.** COMADRE *R* scripts.
**Appendix S5.** Extended literature used in COMADRE 1.0.0.
**Appendix S6.** Funding and extended acknowledgements.
**Appendix S7.** Author contributions.
**Appendix S8.** Supporting information references.
