# Supplementary material for: COMADRE: a global data base of animal demography
Source: J Anim Ecol. 2016 Jan 27;85(2):371–84. doi: 10.1111/1365-2656.12482 (PMC4819704; doi:10.1111/1365-2656.12482)
Supplement: Supplementary file 1 — Appendix S1. Supporting Online Figures. Appendix S2. Constituents of COMADRE. Appendix S3. COMADRE user's guide. Appendix S4. COMADRE R scripts. Appendix S5. Extended literature used in COMADRE 1.0.0. Appendix S6. Funding and extended acknowledgements. Appendix S7. Author contributions. Appendix S8. Supporting information references. [file JANE-85-371-s001.pdf]

Supporting Information for

COMADRE: a global database of animal demography

Roberto Salguero-Gómez, Owen R. Jones, C. Ruth Archer, Christoph Bein, Hendrik de Buhr, Claudia Farack, Fränce Gottschalk, Alexander Hartmann, Anne Henning, Gabriel Hoppe, Gesa Römer, Tara Ruoff, Veronika Sommer, Julia Wille, Jakob Voigt, Stefan Zeh, Dirk Vieregg, Yvonne M. Buckley, Judy Che-Castaldo, David Hodgson, Alexander Scheuerlein, Hal Caswell & James W. Vaupel

Table of Contents

Appendix S1. Supporting figures 3

Online Figure S1 3

Online Figure S2 4

Online Figure S3 5

Appendix S2. Constituents of COMADRE 6

Appendix S3. COMADRE user's guide 8

Appendix S4. COMADRE *R* scripts 9

S4.1. Citation checking 9

S4.2. Taxonomy update 12

S4.3. Plotting a life cycle from a matrix population model 17

S4.4. Simple demographic output for a subset of populations 19

S4.5. Geographic distribution of studied populations 22

S4.6. Ternary plots 26

S4.7. Advanced subsetting and refined searches 35

Appendix S5. Extended literature used in COMADRE v. 1.0.0 37

Appendix S6. Funding and extended acknowledgements 96

Appendix S7. Author contributions 98

Appendix S8. Supporting information references 101

Appendix S1. Supporting figures

Online Figure S1

Full hierarchical organization of the Rdata object `comadre` with an example of output, where *i* = 958 in version 1.0.0 corresponds to the mean population matrix model for the length of the study by Meslow & Keith (1968) on the snowshoe hare. Photo credit: NPS Photo Tim Rains.

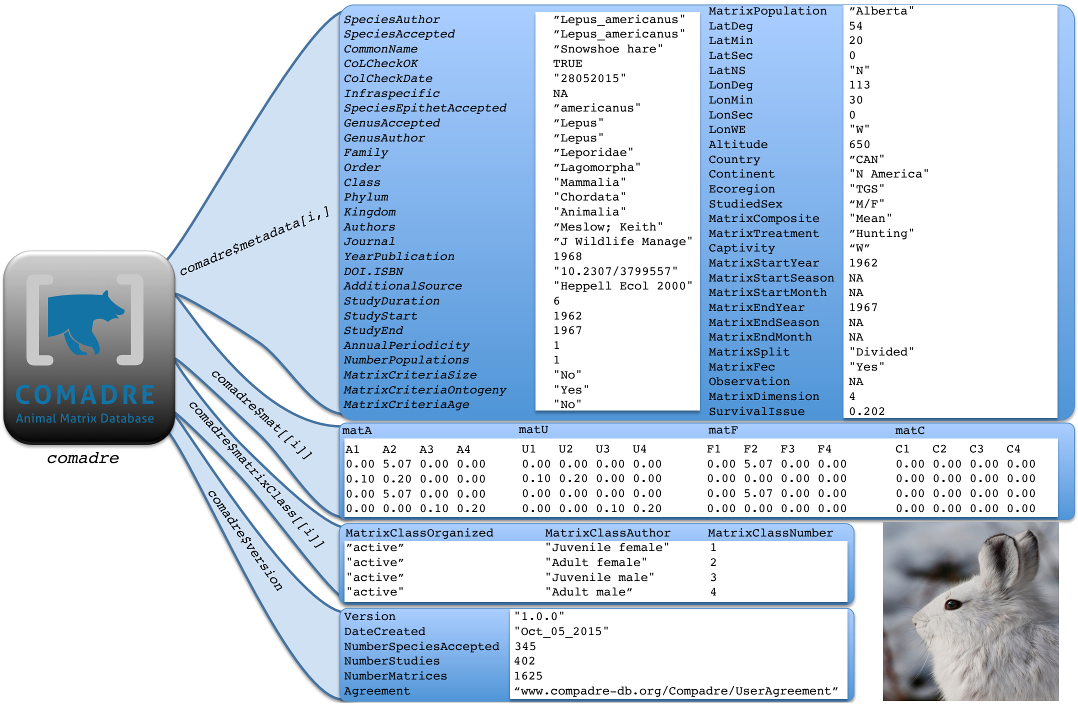

Online Figure S2

Classification of Matrix Population Models (MPMs) in COMADRE according to: **A.** the type of matrix (See *MatrixComposite* in Table 1). **B**. the environmental conditions of studied population (*Captivity*). **C**. the general type of treatment of matrix model under consideration (*MatrixTreatment*). **D.** which sex was modelled (*StudySex*). **E.** whether the matrix ***A*** (equation 2) was split into submatrices ***U***, ***F*** and ***C*** (*MatrixSplit)*. **F**. whether reproduction was modelled (*MatrixFec*). **G**. taxonomic class representation (*Class*). **H**. continent. **I.** Histogram of altitude (m a.s.l.) of studied sites, where negative values correspond to marine ecosystems.

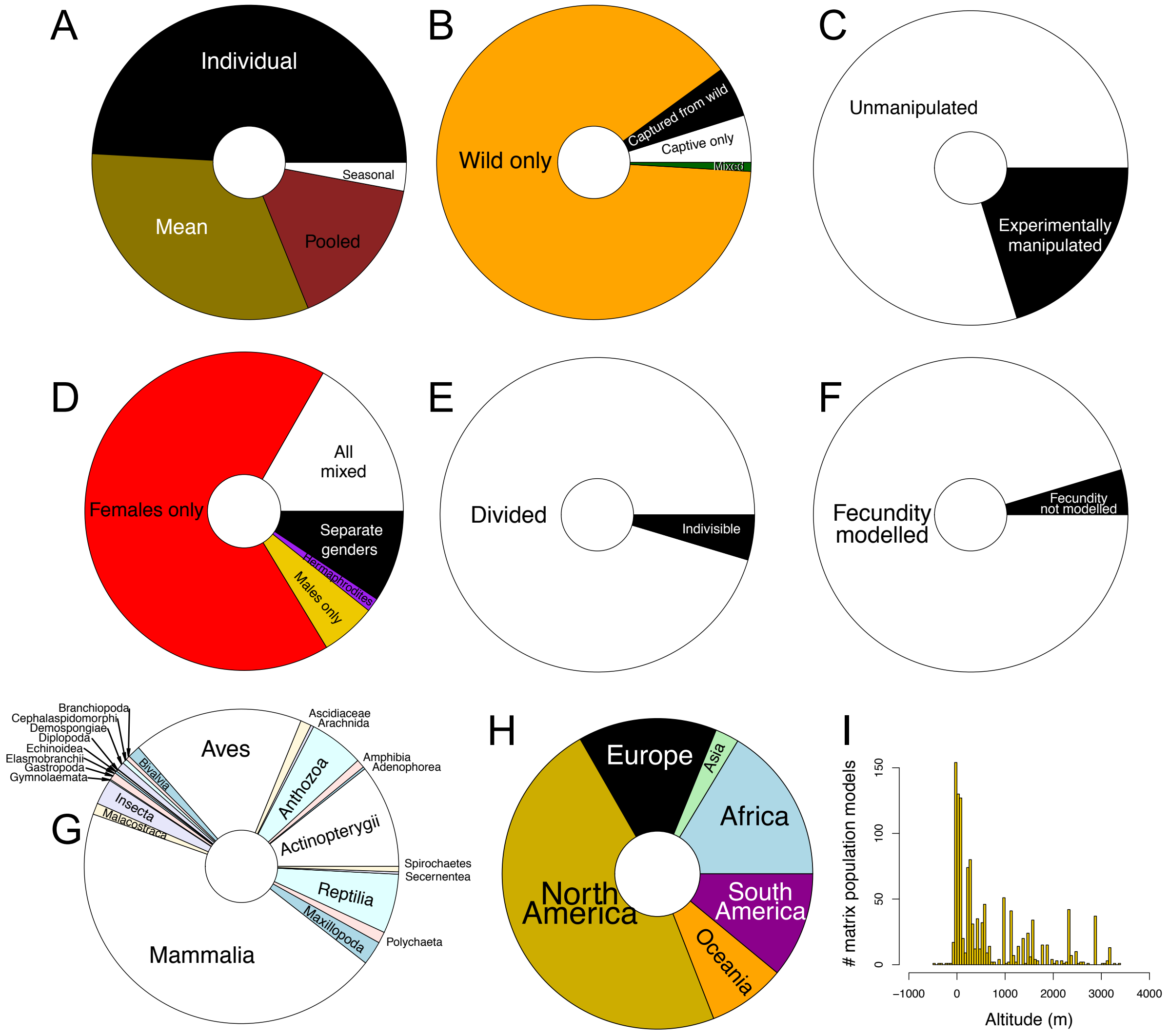

Online Figure S3

Start and end of each study in COMADRE v. 1.0.0.

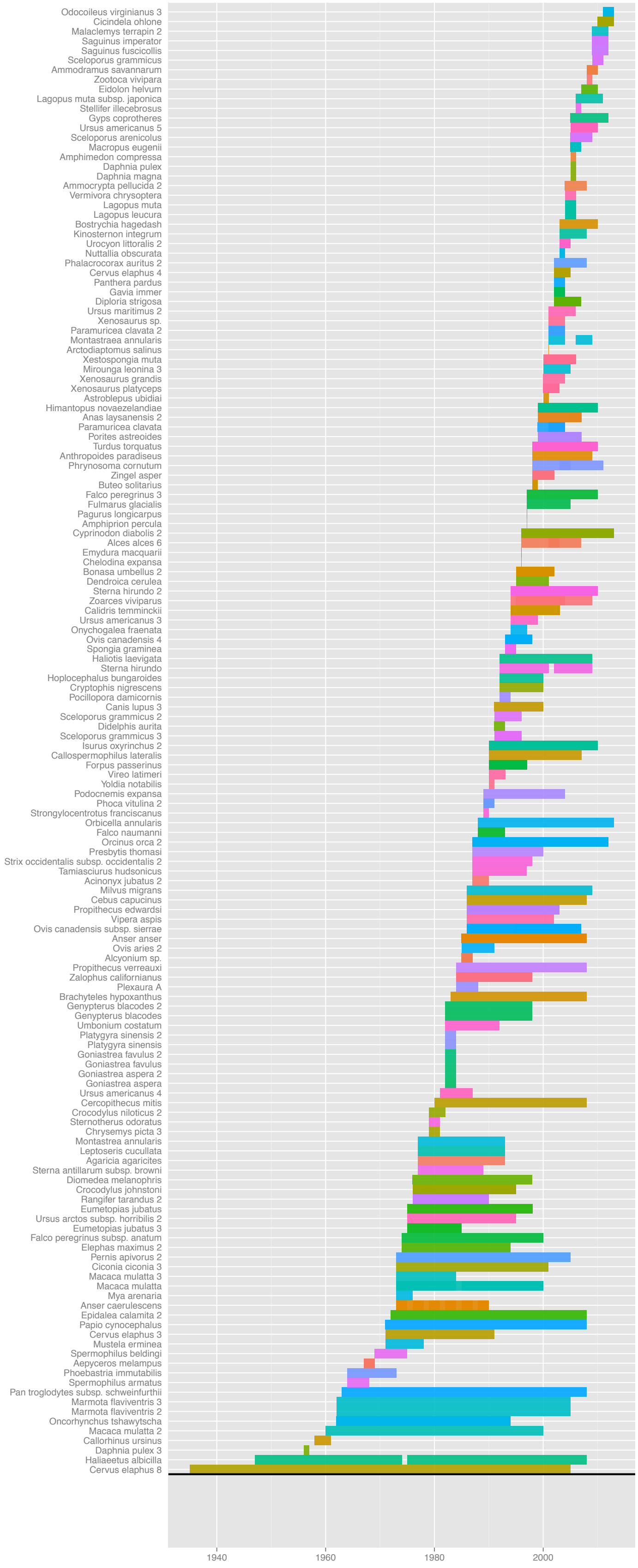

Appendix S2. Constituents of COMADRE

The COMADRE Animal Matrix Database is developed and supported by two committees and a digitization team. Their constituent members, whose tenure is revisited every two years, are located worldwide, assuring that COMADRE reaches all continents, and that published information is sent back to COMADRE from white and gray literature.

	Member	Institution	Country
Core committee	Roberto Salguero-Gómez	University of Queensland<br>Max Planck Institute for Demographic Research (MPIDR)	Australia<br>Germany
	Owen Jones	University of Southern Denmark	Denmark
	Ruth Archer	University of Exeter	UK
	Yvonne Buckley	Trinity College Dublin<br>University of Queensland	Ireland<br>Australia
	Judy Che-Castaldo	National Socio-Environmental Synthesis Center (SESYNC)	USA
	David Hodgson	University of Exeter	UK
	Alexander Scheuerlein	MPIDR	Germany
	Hal Caswell	University of Amsterdam<br>Woods Hole Oceanographic Institute<br>MPIDR<br>University of Amsterdam	The Netherlands<br>USA<br>Germany<br>The Netherlands
	James Vaupel	MPIDR<br>Duke University<br>University of Southern Denmark	Germany<br>USA<br>Denmark
Science committee	Res Altwegg	University of Cape Town	South Africa
	Fernando Colchero	University of Southern Denmark	Denmark
	Dalia Conde	University of Southern Denmark<br>The Centre for Research and Conservation, Antwerp Zoo	Denmark<br>Belgium
	Ming Dong	The Chinese Academy of Sciences	China
	Miguel Franco	University of Plymouth	UK
	Eelke Jongejans	Radboud University	The Netherlands
	Hans de Kroon	Radboud University	The Netherlands
	Jean-Dominique Lebreton	Centre National de la Recherche Scientifique	France
	Jessica Metcalf	Princeton University	UK

	Maile Neel	University of Maryland	USA
	Ingrid Parker	University of California Santa Cruz	USA
	Bernt-Erik Sæther	Norwegian University of Science and Technology	Norway
	Juan Silva	Universidad de los Andes	Venezuela
	Jonathan Silvertown	Open University	UK
	Takenori Takada	Hokkaido University	Japan
	Teresa Valverde	Universidad Nacional Autónoma de México	Mexico
	Luis Antonio Velez-Espino	Fisheries and Oceans Canada	Canada
	Glenda Wardle	University of Sydney	Australia
Digitization team	Christoph Being	MPIDR	Germany
	Hendrik de Buhr	MPIDR	Germany
	Claudia Farack	MPIDR	Germany
	Fränce Gottschalk	MPIDR	Germany
	Alexander Hartmann	MPIDR	Germany
	Anna Henning	MPIDR	Germany
	Gabriel Hoppe	MPIDR	Germany
	Gesa Römer	MPIDR	Germany
	Tara Ruoff	University of Maryland	USA
	Jakob Voigt	MPIDR	Germany
	Julia Wille	MPIDR	Germany
	Stefan Zeh	MPIDR	Germany

Appendix S3. COMADRE user’s guide

The COMADRE Animal Matrix Database user’s guide, which contains all the variables available in COMADRE, the organization of the “*COMADRE v.1.0.0.Rdata* file, and the range of options for each variable, is available here:

http://www.comadre-db.org/

Appendix S4. COMADRE *R* scripts

The COMADRE Animal Matrix Database team is making the following R code available to facilitate comparative demographic research. Various textbooks (Caswell, 2001; Morris & Doak, 2002) and scripts (Cochran & Ellner 1992; Stubben 2007; Stott et al., 2012; Metcalf et al., 2013) can be consulted for further details. The format of the database will likely evolve with time, and some of the functions below may become obsolete. Nevertheless we maintain a set of useful scripts and functions here: https://github.com/jonesor/compadreDB/.

S4.1. Citation checking

The following example illustrates how one can check and find the full references and DOI for the publications used in COMADRE. See also Supporting Information Appendix S4. The code relies on the package `rcrossref` (see https://github.com/ropensci/rcrossref), which queries *CrossRef*, an official Digital Object Identifier (DOI) Registration Agency of the International DOI Foundation. Thus you will first need to load the package (and `install.packages` it if necessary):

```
library(rcrossref)
```

First, set the working directory to where the COMADRE R data object has been saved and load the data:

```
setwd("~/Downloads/")
load("COMADRE_v.1.0.0.RData")
```

The `rcrossref` package has a convenient function, `cr_search_free`, which conducts a free-text search of the CrossRef database. To use it, one needs to provide some query text, so in this case we can simply create a text string by concatenating the

authors, journal and year of publication from COMADRE. For example, to obtain the full reference and DOI for the matrices for the koala, *Phascolarctos cinereus*. Firstly we can identify the pertinent rows in the metadata:

```
id <- which(comadre$metadata$SpeciesAccepted == "Phascolarctos_cinereus")
length(id)

> [1] 5
```

Then we can use this information to obtain the source information (authors, journal and year of publication) for the 5 matrices:

```
temp <- comadre$metadata[id, c("Authors", "Journal", "YearPublication")]
head(temp)

>                                                      Authors   Journal
> 1240       Baxter; McCarthy; Possingham; Menkhorst; McLean Cons Biol
> 1241 Rhodes; Ng; de Villiers; Preece; McAlpine; Possingham Biol Cons
> 1242 Rhodes; Ng; de Villiers; Preece; McAlpine; Possingham Biol Cons
> 1243 Rhodes; Ng; de Villiers; Preece; McAlpine; Possingham Biol Cons
> 1244 Rhodes; Ng; de Villiers; Preece; McAlpine; Possingham Biol Cons
>      YearPublication
> 1240            2006
> 1241            2011
> 1242            2011
> 1243            2011
> 1244            2011
```

Now paste this information together to form a single search string for each matrix. We can optionally ask R to return the unique set of values:

```
x<-apply(temp,1,paste,collapse = " ")
x<-unique(x)
x

> [1] "Baxter; McCarthy; Possingham; Menkhorst; McLean Cons Biol 2006"
> [2] "Rhodes; Ng; de Villiers; Preece; McAlpine; Possingham Biol Cons 201
1"
```

This shows that the data are from two publications for which we can obtain source information via CrossRef. The `cr_search_free` function below, from the library `rcrossref` (above) returns a `data.frame`, with a column called `doi` that contains the returned Digital Object Identifiers for the publications.

```
temp <- cr_search_free(x)
temp$doi

> [1] "http://dx.doi.org/10.1111/j.1523-1739.2006.00378.x"
> [2] "http://dx.doi.org/10.1371/journal.pone.0092430"
```

Armed with the DOI, it is easy to obtain the full title, author list etc. from CrossRef in a range of formats using the function `cr_cn`. This uses the raw DOI, without the `http://dx.doi.org/` prefix. Therefore this prefix must first be stripped from the query using `gsub`.

```
doiValues <- gsub("http://dx.doi.org/","",temp$doi)
cr_cn(dois=doiValues,format = "text", style = "apa")

> [[1]]
> [1] "BAXTER, P. W. J., McCARTHY, M. A., POSSINGHAM, H. P., MENKHORST, P.
 W., & McLEAN, N. (2006). Accounting for Management Costs in Sensitivity A
nalyses of Matrix Population Models. Conservation Biology, 20(3), 893–905.
```

```
 doi:10.1111/j.1523-1739.2006.00378.x"
>
> [[2]]
> [1] "Ng, C. F., Possingham, H. P., McAlpine, C. A., de Villiers, D. L., 
Preece, H. J., & Rhodes, J. R. (2014). Impediments to the Success of Manag
ement Actions for Species Recovery. PLoS ONE, 9(4), e92430. doi:10.1371/jo
urnal.pone.0092430"
```

S4.2. Taxonomy update

The following R code illustrates how to update the taxonomy used in COMADRE using the R package `taxize` (Chamberlain & Szöcs 2013) to query the Catalogue of Life (http://www.catalogueoflife.org) with the species names used by the source publication's original authors and recorded in COMADRE. The function `classification` in `taxize` allows the programmatic querying of databases including the Catalogue of Life. For example:

```
classification("Daphnia magna", db = 'col')

>

> Retrieving data for taxon 'Daphnia magna'

> $`Daphnia magna`
>             name     rank                               id
> 1       Animalia  Kingdom 5ede24b0534ebd5e1f552d5b9f874a6a
> 2     Arthropoda   Phylum 89ac18bfcf1654a9662a600ba06bb494
> 3   Branchiopoda    Class 1793fd966478a2d1c959221e2a711569
> 4    Diplostraca    Order 04df00f2834eac8dc22966b299226a13
> 5   Not assigned   Family                         24298066
```

```
> 6       Daphnia    Genus 1acee7e4189fdad4c8abf00900254b85
> 7  Ctenodaphnia Subgenus                          24532001
> 8 Daphnia magna  Species e44e0a35c2cdb085859074a9fe73abdf
>
> attr(,"class")
> [1] "classification"
> attr(,"db")
> [1] "col"
```

One can capitalize on this for COMADRE. First, load the data:

```
load("COMADRE_v.1.0.0.RData")
```

Then, load the required package, `taxize`.

```
library(taxize)
```

Next, make a new vector called `SpeciesBinomial` by concatenating the accepted genus (`GenusAccepted`) and accepted species epithet (`SpeciesEpithetAccepted`) together. This is necessary, rather than simply using `SpeciesAccepted`, because `SpeciesAccepted` retains the infra-specific information, which is not used by the following code:

```
comadre$metadata$SpeciesBinomial <-
paste(comadre$metadata$GenusAccepted, comadre$metadata$SpeciesEpithetAccep
ted)
```

Some species do not have an epithet (e.g. *Tribolium* sp.), and for these the epithet is listed as `NA`. Therefore, to search the Catalogue of Life effectively, the `NA` needs to be removed using `gsub`:

```
comadre$metadata$SpeciesBinomial <- gsub("NA", "",
                                          comadre$metadata$SpeciesBinomial)
```

Because species appear in the database numerous times, it is advisable for efficiency reasons to make a unique subset of the data:

```
temp <- unique(comadre$metadata[, c("SpeciesBinomial",
"GenusAccepted", "Family", "Order", "Class", "Phylum", "Kingdom")])
```

This dataset is still quite large (334 rows), so here I will just obtain the information for the first 5 rows.

```
temp <- temp[1:5,]
```

This is accomplished using the `classification` function, which repeatedly queries the Catalogue of Life for each entry. Note that whenever there is an uncertainty as to which species is intended, the software prompts the user to select a species from a list.

```
x <- classification(temp$SpeciesBinomial,db='col')

>

> Retrieving data for taxon 'Acipenser fulvescens'

>

>

> Retrieving data for taxon 'Acipenser transmontanus'

>

>

> Retrieving data for taxon 'Ambloplites rupestris'

>

>
```

```
> Retrieving data for taxon 'Ammocrypta pellucida'
>
>
> Retrieving data for taxon 'Amphiprion percula'
```

The object produced by this code is a `list` with elements corresponding to each species. Thus `x[[1]]` will return the first species etc. One can also address the list by element name, which is defined by the query that was submitted e.g. `x[["Acipenser fulvescens"]]`

```
x[[1]]

>                   name    rank                               id
> 1             Animalia Kingdom 5ede24b0534ebd5e1f552d5b9f874a6a
> 2             Chordata  Phylum 4313bc7637e1fc1feb316a4dea2b668b
> 3       Actinopterygii   Class 655300e5b96a3dc5e76e02175c3aa191
> 4     Acipenseriformes   Order 1b6074738c3c562418eee137a0369bdb
> 5        Acipenseridae  Family 5a7e5d2984e865f1aff640138c760d04
> 6            Acipenser   Genus b506984ec1a4c2f6191b842dcbce8fa7
> 7 Acipenser fulvescens Species ebc713afa86464a0cfb313955a1db149
```

Thus, to acquire the taxonomic Order of the species of interest one would use:

```
x[[1]]$name[x[[1]]$rank == "Order"]

> [1] "Acipenseriformes"
```

One can easily run through this list object in a loop to compare the information held in COMADRE, with that produced by the query to Catalogue of Life. For example, to check the Orders of the five species held in `temp`:

```
for(i in 1:nrow(temp)){

n <- temp$SpeciesBinomial[i]

dat <- x[[n]]

CoL_Order <- dat$name[dat$rank == "Order"]

if(temp$Order[i]==CoL_Order){temp$OrderOK[i] <- "OK"} else {temp$OrderOK

[i] <- "Differs!"}

}
```

Which will add an extra column called `OrderOK` to the `temp` data frame:

```
temp

>             SpeciesBinomial GenusAccepted        Family             Order

> 1      Acipenser fulvescens     Acipenser Acipenseridae Acipenseriformes

> 14 Acipenser transmontanus     Acipenser Acipenseridae Acipenseriformes

> 15    Ambloplites rupestris   Ambloplites Centrarchidae      Perciformes

> 18     Ammocrypta pellucida    Ammocrypta       Percidae      Perciformes

> 20       Amphiprion percula    Amphiprion Pomacentridae      Perciformes

>             Class   Phylum  Kingdom OrderOK

> 1  Actinopterygii Chordata Animalia      OK

> 14 Actinopterygii Chordata Animalia      OK

> 15 Actinopterygii Chordata Animalia      OK

> 18 Actinopterygii Chordata Animalia      OK

> 20 Actinopterygii Chordata Animalia      OK
```

One could obviously repeat this for each part of the taxonomy.

S4.3. Plotting a life cycle from a matrix population model

This example plots a life cycle diagram with the stages and transitions of a given matrix chosen from the `comadre` database. It will call the R function `plotLifeCycle` from the COMADRE github repository. This works well with matrices of relatively low dimensionality (≤ 7), and where not many transitions are depicted. The function is based on the library `DiagrammeR`, so this needs to be called first:

```
library(DiagrammeR)
```

Let us first consider plotting the lifecycle for one of the species containing the word "lion" in the common name used by the author(s) in the original source used in the COMADRE database. As before, first load `comadre`:

```
load(file ="COMADRE_v.1.0.0.RData")
```

To find the species with the word `lion` in their common name, we use the function `grep`.

```
lions <- grep("lion",comadre$metadata$CommonName)
unique(comadre$metadata$CommonName[lions])

> [1] "Common lionfish"
> [2] "Red lionfish"
> [3] "Northern sea lion; Steller sea lion"
> [4] "New Zealand sea lion"
> [5] "California sea lion"
```

Unfortunately, no actual lion (*Panthera leo*) has been included in this version of COMADRE, but there are plenty of other 'lions' in it. We will plot the life cycle of the red lionfish (*Pterois volitans*):

```
matNum <- which(comadre$metadata$CommonName == "Red lionfish")

matNum


> [1] 151
```

```
sp <- gsub("_"," ",comadre$metadata$SpeciesAccepted[matNum])
sp

> [1] "Pterois volitans"
```

The matrix **A** and the stages of this study are:

```
matA <- comadre$mat[[matNum]]$matA
matA

>          A1    A2     A3
> [1,] 0e+00 0.000 35.315
> [2,] 3e-05 0.777  0.000
> [3,] 0e+00 0.071  0.949

stages <- comadre$matrixClass[[matNum]]$MatrixClassAuthor
stages

> [1] "Larvae"                 "Juvenile (20 - 174 mm)"
> [3] "Adult (> 174 mm)"
```

To plot its lifecycle, source the function *plotLifeCycle* from the GitHub repository of the database using `source_url` function from the `devtools` library:

```
require(devtools)
> Loading required package: devtools
source_url("https://raw.githubusercontent.com/jonesor/compadreDB/master/Functions/plotLifeCycle.R")
> SHA-1 hash of file is 9c8b033012c5de1418bf653804811179fb7cda5b
plotLifeCycle(matA, title = "Red lionfish")
```

The resulting life cycle is:

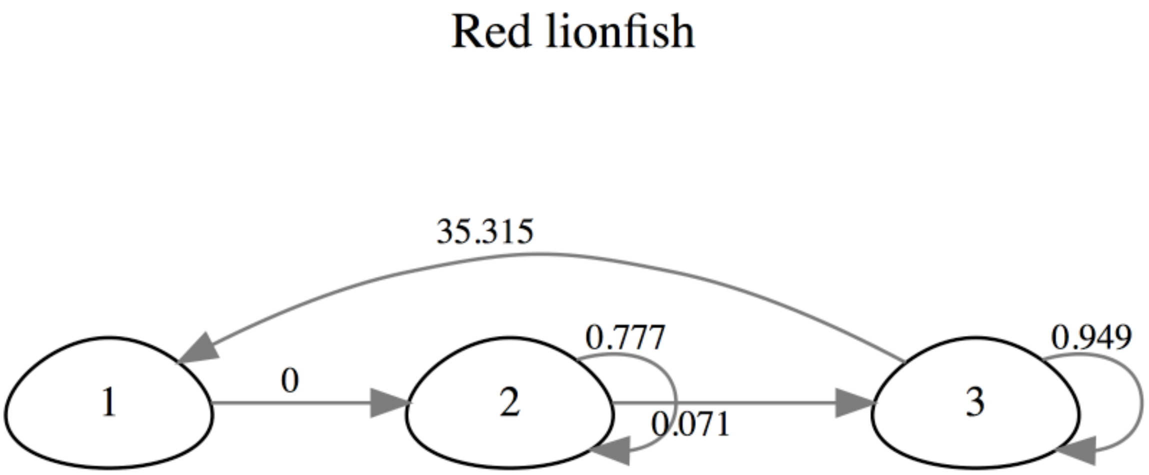

S4.4. Simple demographic output for a subset of populations

This example produces some basic output such as the population growth rate (λ) and damping ratio (Caswell 2001) for a subset of species and populations given some selection criteria.

First we can `subset` the database to the data of interest: only mean matrices for bony fish from studies of three years duration or longer, and with a matrix dimension of three or greater.

```
tempMetadata <- subset(comadre$metadata,
                       MatrixComposite == "Mean" &
                         Class == "Actinopterygii" &
                         StudyDuration >= 3 &
                         MatrixDimension > 3)
```

The row names from the subsetted dataframe can now be used to subset the entire `comadre` database using the function `subsetDB`, which is available as part of this supplementary information (Run it in Appendix 4.7 below).

```
id <- as.numeric(rownames(tempMetadata))

x<-subsetDB(comadre,id)
```

The object `x` is now a subsetted version of the `comadre` database object that contains only the matrices that match the search criteria.

These matrices can now be analyzed by applying functions in a loop, or by using `lapply`. For example, to calculate population growth rate and damping ratio for the subset of matrices, we can first create an empty `data.frame` to accommodate the output:

```
output <- data.frame(lambdas = rep(NA, length(x$mat)),

                     damps = rep(NA, length(x$mat)))
```

then use the functions in `popbio` package to derive demographic output (you may need to install the package first).

```
library(popbio)

> Loading required package: quadprog

for (i in 1:length(x$mat)){

    output$lambdas[i] <- Re(eigen(x$mat[[i]]$matA)$value)[1]

    output$damps[i] <- damping.ratio(x$mat[[i]]$matA)

}
```

Let's take a look at the output:

```
output
```

```
>       lambdas    damps
> 1  1.5061504 1.303947
> 2  0.9704512 1.468910
> 3  0.9529318 1.127114
> 4  0.9091956 1.031997
> 5  0.9027406 1.027850
> 6  0.9140835 1.035914
> 7  0.9999592 1.005268
> 8  1.1102061 1.310725
> 9  1.3618336 1.293028
> 10 1.2283756 1.380559
> 11 1.1321531 1.288657
```

These parameters are presented in the same order as the `metadata` so we could also add the species name to this `data.frame`:

```
data.frame(Species = x$metadata$SpeciesAccepted,output)

>                               Species   lambdas    damps
> 1                 Ammocrypta_pellucida 1.5061504 1.303947
> 2                  Genypterus_blacodes 0.9704512 1.468910
> 3                  Genypterus_blacodes 0.9529318 1.127114
> 4             Oncorhynchus_tshawytscha 0.9091956 1.031997
> 5             Oncorhynchus_tshawytscha 0.9027406 1.027850
> 6             Oncorhynchus_tshawytscha 0.9140835 1.035914
> 7  Sprattus_sprattus_subsp._balticus 0.9999592 1.005268
> 8                    Zoarces_viviparus 1.1102061 1.310725
> 9                    Zoarces_viviparus 1.3618336 1.293028
```

```
> 10                   Zoarces_viviparus 1.2283756 1.380559
> 11                   Zoarces_viviparus 1.1321531 1.288657
```

Now one can plot the population growth rates and damping ratios derived from these matrices. In this plot, the vertical, dashed red line indicates population growth rate = 1 (or log (λ) = 0)

```
par(mfrow = c(1,2))
hist(log(output$lambdas),
     xlab = "Log population growth rate",
     col = "gold", main = "")
abline(v=0,col = "red", lwd = 4, lty = 3)
hist(output$damps, xlab = "Damping ratio",
     col = "brown", main = "")
```

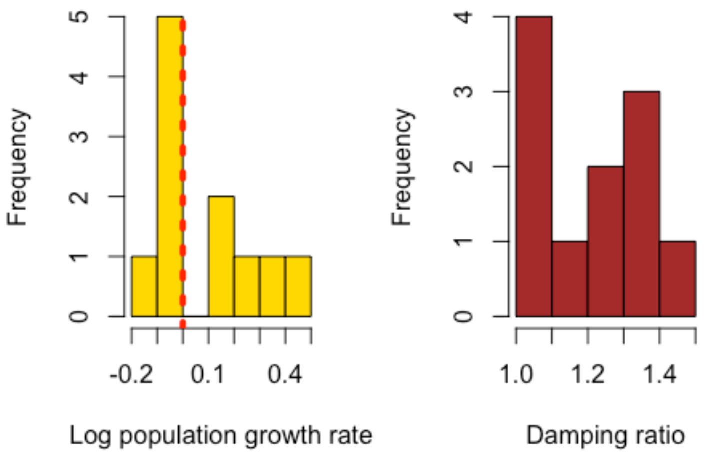

S4.5. Geographic distribution of studied populations

This example produces on a world map the viability (population growth rate $\lambda > 1$, $\lambda = 1$, $\lambda < 1$) of a subset of studied populations given some selection criteria, and color-codes the location of each population according to the value of λ.

First, subset mean matrices for all Carnivora in the wild in the Northern hemisphere, with no issues for survival (no stage-specific survival >1), for which matrices have been split into **A** = **U** + **F** + **C**, and for which reproduction was explicitly modeled.

```
tempMetadata <- subset(comadre$metadata,
                       MatrixComposite == "Mean" &
                         Order == "Carnivora" &
                         MatrixCaptivity == "W" &
                         LatNS == "N" &
                         SurvivalIssue < 1 &
                         MatrixSplit == "Divided" &
                         MatrixFec == "Yes")
```

Now, use the row names from the subsetted dataframe to subset the matrices. The function subsetDB used here is described below (Run it in Appendix 4.7 below).

```
id <- as.numeric(rownames(tempMetadata))
x <- subsetDB(comadre,id)
```

The object `x` is now a section of the `comadre` database object that contains only the matrices that match the search criteria. To calculate population growth rate for the subset matrices, first create an empty `data.frame` to accommodate the output:

```
output <- data.frame(lambdas = rep(NA, length(x$mat)))
```

Now create dummy variables to convert geographic information to be plotted on the map (below):

```
x$metadata$LAT <- NA
x$metadata$LON <- NA
```

```
for(i in 1:nrow(x$metadata)){
  if(x$metadata$LatNS[i] == "S") {
    x$metadata$LatDeg[i] <- -x$metadata$LatDeg[i]
  }
  if(x$metadata$LonWE[i] == "W") {
    x$metadata$LonDeg[i] <- -x$metadata$LonDeg[i]
  }
  x$metadata$LAT[i] <- x$metadata$LatDeg[i] +
    x$metadata$LatMin[i]/60 + x$metadata$LatSec[i]/3600
  x$metadata$LON[i] <- x$metadata$LonDeg[i] +
    x$metadata$LonMin[i]/60 +
    x$metadata$LonSec[i]/3600
}
```

Create an empty variable to accommodate output from lambda calculations:

```
x$metadata$lambdas <- NA
```

Then, create a `for` loop to examine each matrix in turn. Here it may be advisable to use the function `tryCatch` as a wrapper to cope with the situation if/when the function in the loop fails:

```
for (i in 1:length(x$mat)){
  tryCatch({
    x$metadata$lambdas[i] <- Re(eigen(x$mat[[i]]$matA)$value)[1]
  }, error = function(e){})
}
```

Next, create a vector of color hex codes that can be applied according to the estimate of λ. This is done using the `colorRampPalette` function to go from green for

high values of λ, to red for low values of λ. Here `paste` is used to append a value of `90` to the hex codes to allow transparency for aesthetic reasons.

```
rampfunc <- colorRampPalette(c("green", "red"))
colVect <- rampfunc(100)
colVect <- paste(colVect,"90",sep="")
s1 <- seq(min(x$metadata$lambdas,na.rm=TRUE),max( x$metadata$lambdas,na.rm
=TRUE),length.out = 100)
```

It is necessary to load the `maps` package (and install it if necessary). Then, plot the world map, and overlay the points from our data, color-coded by value of λ. In this case, the points are jittered slightly to improve visibility of nearby populations.

```
library(maps)
map("world", col = "gray", fill = TRUE, bg = "light blue",
    xlim=c(-175,176),ylim=c(-60, 85), border = "white")

points(jitter(x$metadata$LON,amount=0.6),jitter(x$metadata$LAT,amount = 0.
6),col = colVect[findInterval(x$metadata$lambdas,s1)],cex=2,pch=16)
```

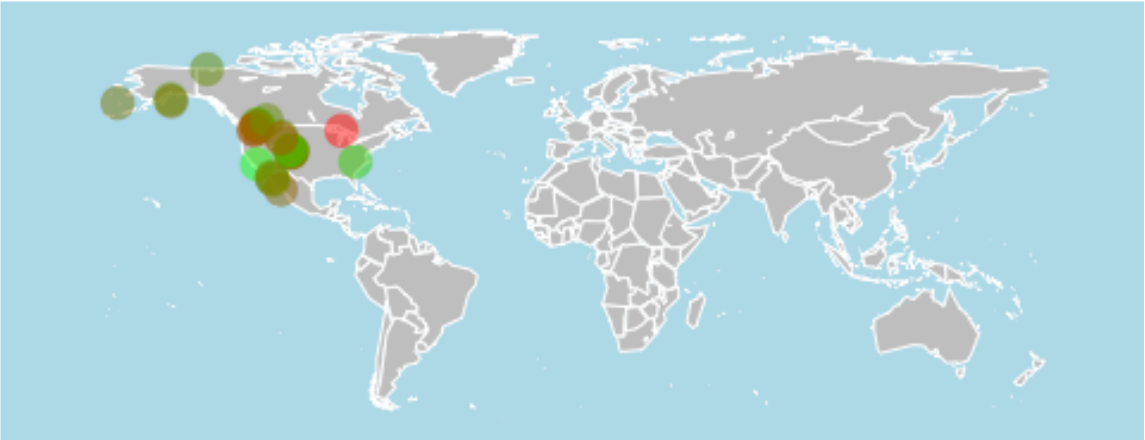

S4.6. Ternary plots

Here we illustrate how to produce a ternary plot *a la* Silvertown *et al* (1993) with various life history traits such as population growth rate (λ),mean life expectancy (η_e), or reactivity ($||\hat{A}||_1$) as the "fourth" dimension. We will use Caswell (2001) formulation of mean life expectancy from the fundamental matrix (***N***), and the packages `popdemo` for the reactivity:

```
load("COMADRE_v.1.0.0.RData")
require(popdemo)

> Loading required package: popdemo
```

This is the function to calculate mean life expectancy from Caswell (2001):

```
meanLifeExpectancy <- function(matU = matU, startLife = 1){
  uDim=dim(matU)[1]
  N = solve(diag(uDim[startLife])-matU)
  eta = colSums(N)[startLife]
  return(eta)
}
```

As an example for the chosen data, subset COMADRE to studies with a matrix dimension ≥ 3, that represent mean, unmanipulated conditions duration > 3 years, where sexual reproduction has been modeled explicitly, the matrices are split into U, F and C, and there are no issues with stage-specific survival >1

```
tempMetadata <- subset(comadre$metadata, MatrixDimension >= 3 &
                       MatrixComposite == "Mean" &
                       MatrixTreatment == "Unmanipulated" &
```

```
        StudyDuration > 3 & MatrixFec == "Yes" &

        MatrixSplit == "Divided" & SurvivalIssue < 1)
```

Use the row names from the subsetted dataframe to subset the matrices.

```
keep <- as.numeric(rownames(tempMetadata))
```

Define the object containing MPMs in the same order that their metadata appears in `tempMetadata`.

```
tempMat <- comadre$mat[keep]
```

These MPMs can now be analyzed by applying functions in a loop, or by using `lapply`. To calculate elasticities, population growth rate, reactivity and mean life expectancy for the subset matrices, first crate a dummy variable to accommodate the output.

```
output <- data.frame(species= rep(NA, length(tempMat)),
                      lambdas = rep(NA, length(tempMat)),
                      eta = rep(NA, length(tempMat)),
                      react = rep(NA, length(tempMat)),
                      EStasis = rep(NA, length(tempMat)),
                      EProgression = rep(NA, length(tempMat)),
                      ERetrogression = rep(NA, length(tempMat)),
                      EFecundity = rep(NA, length(tempMat)),
                      EClonality = rep(NA, length(tempMat)))
```

Use the following function to calculate element-level perturbations:

```
matrixElementPerturbation <- function(matU, matF, matC=NULL,pert=0.001){
  matA=matU+matF+matC
  aDim=dim(matA)[1]
```

```
  fakeA=matA
  sensA=elasA=matrix(NA,aDim,aDim)
  lambda=Re(eigen(matA)$values[1])
  propU=matU/matA
    propU[is.nan(propU)]=NA
    propProg=propRetrog=propU
    propProg[upper.tri(propU,diag=T)]=NA
    propRetrog[lower.tri(propU,diag=T)]=NA
    propStasis=matrix(diag(aDim)*diag(propU),aDim,aDim)
  propF=matF/matA
    propF[is.nan(propF)]=NA
  propC=matC/matA
    propC[is.nan(propC)]=NA
  for (i in 1:aDim){
    for (j in 1:aDim){
        fakeA=matA
        fakeA[i,j]=fakeA[i,j]+pert
        lambdaPert=eigen(fakeA)$values[1]
        sensA[i,j]=(lambda-lambdaPert)/(matA[i,j]-fakeA[i,j])
    }
  }
  sensA=Re(sensA)
  elasA=sensA*matA/lambda
  out = data.frame("SStasis"=NA,"SProgression"=NA,"SRetrogression"=NA,"SFe
cundity"=NA,"SClonality"=NA, "EStasis"=NA,"EProgression"=NA,"ERetrogressio
n"=NA,"EFecundity"=NA,"EClonality"=NA)
    out$SStasis=sum(sensA*propStasis,na.rm=T)
```

```
    out$SRetrogression=sum(sensA*propRetrog,na.rm=T)

    out$SProgression=sum(sensA*propProg,na.rm=T)

    out$SFecundity=sum(sensA*propF,na.rm=T)

    out$SClonality=sum(sensA*propC,na.rm=T)

    out$EStasis=sum(elasA*propStasis,na.rm=T)

    out$EProgression=sum(elasA*propProg,na.rm=T)

    out$ERetrogression=sum(elasA*propRetrog,na.rm=T)

    out$EFecundity=sum(elasA*propF,na.rm=T)

    out$EClonality=sum(elasA*propC,na.rm=T)

  return(out)

}
```

Now we can use a loop to examine each matrix:

```
for (i in 1:length(tempMat)){

 tryCatch({ #This command allows for the whole loop to run even with mista
kes. Users are cautioned to examine the output with the command ‘warnings
()’

    matA=tempMat[[i]]$matA

    matU=tempMat[[i]]$matU

    matF=tempMat[[i]]$matF

    matC=tempMat[[i]]$matC

    output$species[i] <- tempMetadata$SpeciesAuthor[i]

    output$lambdas[i] <- max(Re(eigen(matA)$value))

    output$eta[i] = meanLifeExpectancy(matU=matU,startLife=1)

    output$react[i] <- reactivity(matA)

    output[i,c("EStasis","EProgression","ERetrogression","EFecundity","ECl
onality")]=matrixElementPerturbation(matU=matU,matF=matF,matC=matC)[6:10]
```

```
      }, error = function(e){})
}
```

Now, we can group the elasticities of population growth rate to various demographic processes into three main axes, one for stasis and retrogression, another for progression, and another for sexual and clonal reproduction.

```
output$S=output$EStasis+output$ERetrogression
output$G=output$EProgression
output$R=output$EFecundity+output$EClonality
```

Scale the coordinates of each point to 1 - this is necessary due to possible rounding errors, although note that the function to create the ternary plot below can do this automatically with the argument `scale`.

```
output$S=output$S/rowSums(output[,c("S","G","R")])
output$G=output$G/rowSums(output[,c("S","G","R")])
output$R=output$R/rowSums(output[,c("S","G","R")])
```

Eliminate the couple of MPMs where the code did not run correctly, as it produced all NAs:

```
output=output[-which(is.na(output$eta)),]
```

Plot the locations of the chosen matrices in a preliminary ternary plot, for which the libraries `vcd` and `scales` will be necessary.

```
require(vcd)
require(scales)
```

```
ternaryplot(output[,c("R","S","G")],scale=1,col=alpha("gray80",0.7),bg="bl
ack", dimnames=c("Stasis","Growth","Reproduction"),dimnames_position="edge
", main="Preliminary plot")
```

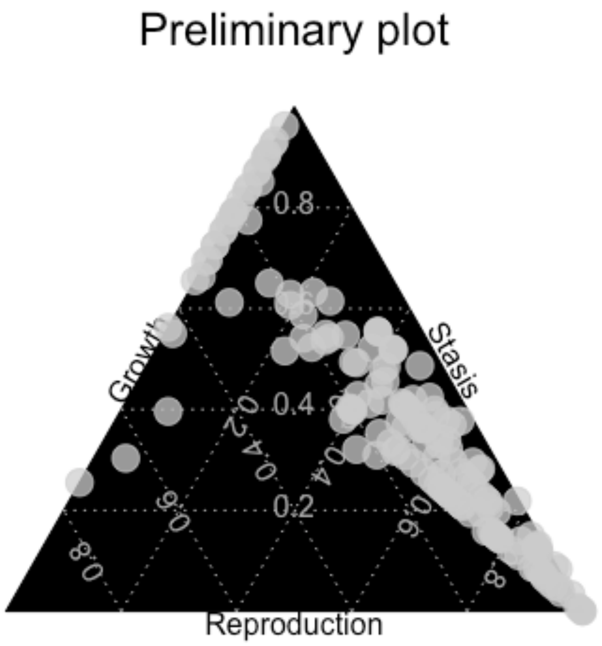

Color-code the points in the ternary plot according to range of lambda, eta and reactivity, respectively, on different plots:

```
lambdaData=output[which(log(output$lambdas)<=2),] #Excluding high lambdas
x_norm=log(lambdaData$lambda)
x_norm = (lambdaData$lambda - min(lambdaData$lambda)) / (max(lambdaData$la
mbda) - min(lambdaData$lambda))
col_fun <- colorRamp(c("white","yellow","orange","red","dark red"))
rgb_cols <- col_fun(x_norm)
colsLambda <- rgb(rgb_cols, maxColorValue = 256)


etaData=output
etaData$etalog=log(etaData$eta)
x_norm = (etaData$etalog - min(etaData$etalog)) / (max(etaData$etalog) - m
```

```
in(etaData$etalog))
rgb_cols <- col_fun(x_norm)
colsEta <- rgb(rgb_cols, maxColorValue = 256)


reactData=output[which(log(output$react)<=5),]
reactData$reactlog=log(reactData$react)
x_norm = (output$react - min(output$react)) / (max(output$react) - min(out
put$react))
rgb_cols <- col_fun(x_norm)
colsReact <- rgb(rgb_cols, maxColorValue = 256)
```

And next plot all three ternary plots. The legend will need the library `fields`.

```
library(fields)
  zr <- range(c(lambdaData$lambda,na.rm=T))
  colCode <- colorRampPalette(c("white","yellow","orange","red","dark red
"))(n = 999)
  image.plot(legend.only=TRUE, zlim= zr, col=colCode, smallplot=c(.75,.8, .
5,.75),cex.axis=0.2)

ternaryplot(lambdaData[,c("R","S","G")],scale=1,col=alpha(colsLambda,0.7),
bg="black", newpage=F, dimnames=c("Stasis","Growth","Reproduction"),dimnam
es_position="edge", main=expression(paste("Population growth rate - ", lam
bda)))
```

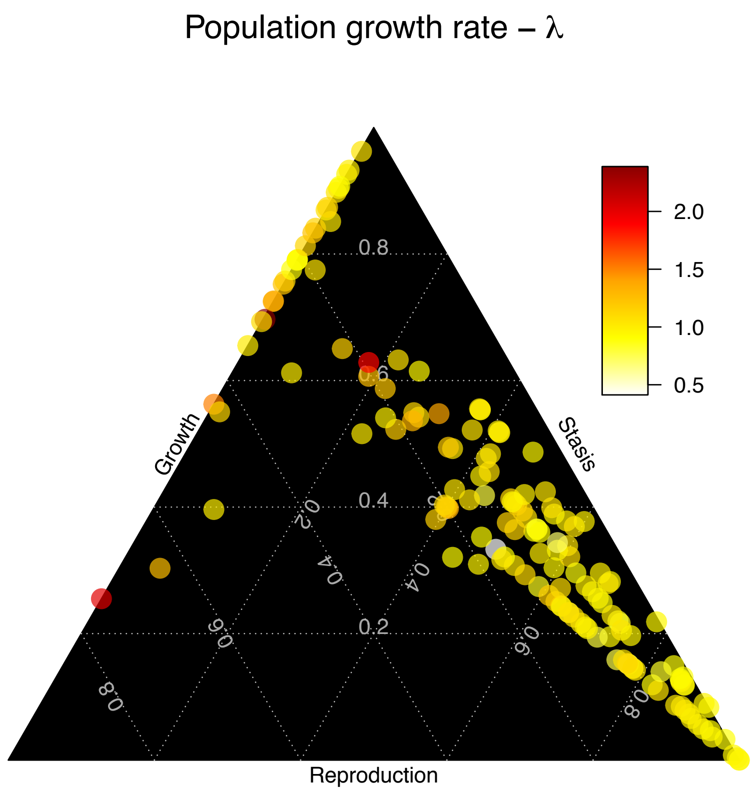


```
zr <- range(c(etaData$eta,na.rm=T))
colCode <- colorRampPalette(c("white","yellow","orange","red","dark red"))
(n = 999)
image.plot(legend.only=TRUE, zlim= zr, col=colCode, smallplot=c(.75,.8, .
5,.75),cex.axis=0.2)



ternaryplot(etaData[,c("R","S","G")],scale=1,col=alpha(colsEta,0.7),bg="bl
ack", newpage=F, dimnames=c("Stasis","Growth","Reproduction"),dimnames_pos
ition="edge", main=expression(paste("Mean life expectancy - ", eta["e"])))
```

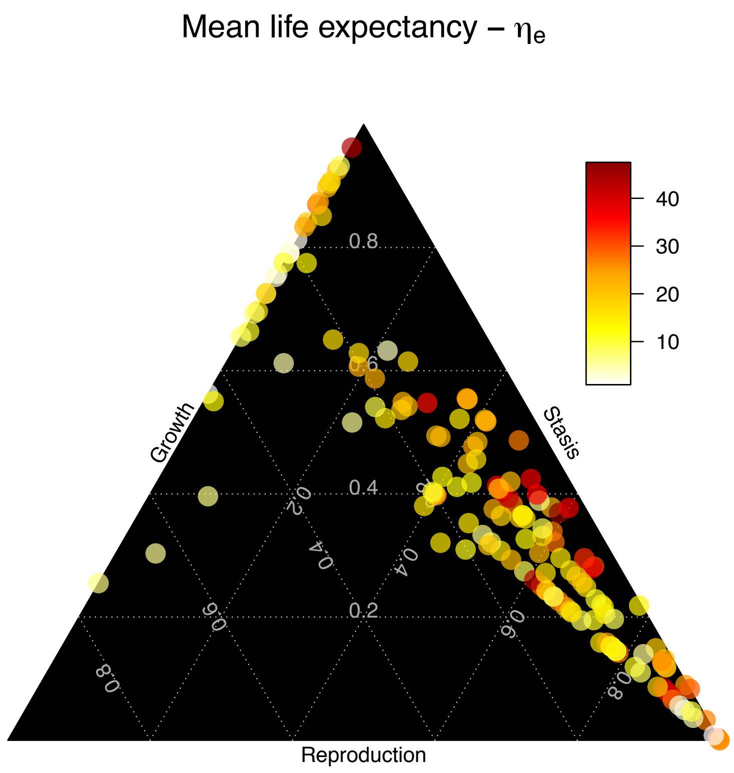


```
zr <- range(c(reactData$react,na.rm=T))

colCode <- colorRampPalette(c("white","yellow","orange","red","dark red"))

(n = 999)

image.plot(legend.only=TRUE, zlim= zr, col=colCode, smallplot=c(.75,.8, .

5,.75),cex.axis=0.2)

zr <- range(c(reactData$react,na.rm=T))

colCode <- colorRampPalette(c("white","yellow","orange","red","dark red"))

(n = 999)

image.plot(legend.only=TRUE, zlim= zr, col=colCode, smallplot=c(.75,.8, .

5,.75),cex.axis=0.2)
```

```
ternaryplot(reactData[,c("R","S","G")],scale=1,col=alpha(colsReact,0.7),bg
="black", newpage=F, dimnames=c("Stasis","Growth","Reproduction"),dimnames
_position="edge",main=expression(paste("Reactivity - ||", hat(A),"||"
[1])))
```

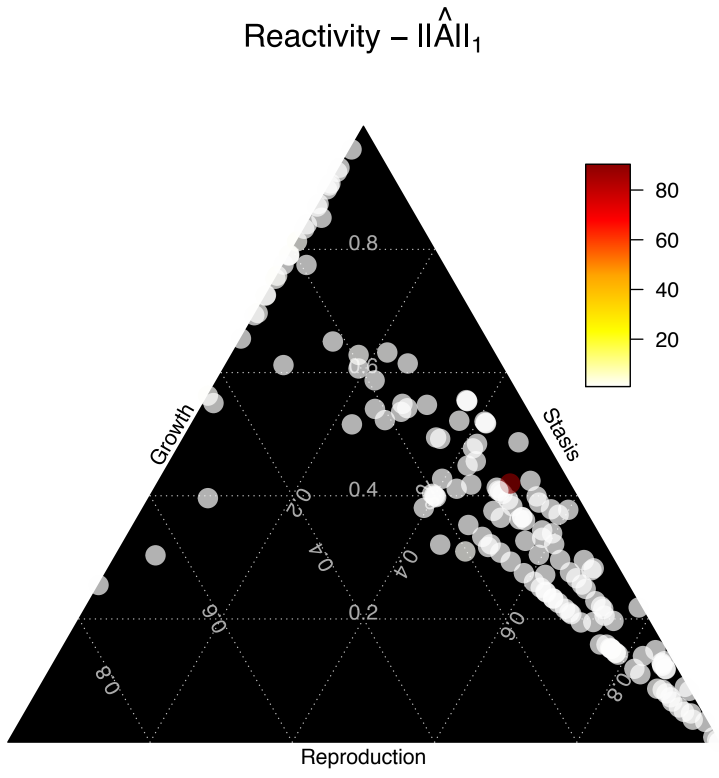

S4.7. Advanced subsetting and refined searches

The `subsetDB` function is a helper function that subsets the entire `comadre` database in a rather fast and convenient manner. There are two arguments: `db` and `sub`. Argument `db` points to the database that you want to subset (usually `comadre` it can also work with `compadre`; Salguero-Gómez *et al.* 2015), while `sub` is an integer vector of row numbers from the database metadata - usually based on some search criteria.

```
subsetDB <- function(db=comadre,sub=1:100){
    subsetID <- sub
    ssdb <- db
```

```
ssdb$metadata <- ssdb$metadata[subsetID,]

ssdb$mat <- ssdb$mat[subsetID]

ssdb$matrixClass <- ssdb$matrixClass[subsetID]

return(ssdb)

}
```

Appendix S5. Extended literature used in COMADRE v. 1.0.0

Below we provide the full list of citations used in the information compiled in the first release of COMADRE (version 1.0.0). Users of these materials are strongly encouraged to credit the work of the specific studies by citing the publications whose information they may use.

The name "*SpeciesAuthor*" corresponds to the exact taxonomic name used by the author in the publication, as detailed in Table 1, with a sequential numerical suffix if more than one study exists for the same species (e.g. *Ursus_americanus, Ursus_americanus_2*). *NA* in citation refers to a secondary citation (see Table 1 in manuscript).

	SpeciesAuthor	SpeciesAccepted	CommonName	Authors	Journal	YearPublication	DOI/ISBN
1	*Acipenser_fulvescens*	*Acipenser_fulvescens*	Lake sturgeon	Velez-Espino; Koops	N Am J Fish Manag	2009	10.1577/M08-034.1
2	*Acipenser_fulvescens_2*	*Acipenser_fulvescens*	Lake sturgeon	Velez-Espino; Koops	Ecol Model	2012	10.1016/j.ecolmodel.2012.09.022
[illegible]	*Acipenser_transmo*[illegible]	*Acipenser_transmont*[illegible]	[illegible]	Velez-Espino; [illegible]	Ecol [illegible]	[illegible]	10.1016/j.ecolmo[illegible]

	ris_2				Thesis		
5	*Ammocrypta_pellucida*	*Ammocrypta_pellucida*	Eastern sand darter	Velez-Espino; Koops	Ecol Model	2012	10.1016/j.ecolmodel.2012.09.022
6	*Ammocrypta_pellucida_2*	*Ammocrypta_pellucida*	Eastern sand darter	Finch; Velez-Espino; Doka; Power; Koops	Fish & Oceans Can	2011	None
7	*Amphiprion_percula*	*Amphiprion_percula*	Orange clown anemonefish	Buston; Garcia	J Fish Biol	2007	10.1111/j.1095-8649.2007.01445.x
8	*Astroblepus_ubidiai*	*Astroblepus_ubidiai*	Andean catfish	Velez-Espino	Ecol Freshwater Fish	2005	10.1111/j.1600-0633.2005.00084.x
9	*Brachyrhaphis_rhabdophora*	*Brachyrhaphis_rhabdophora*	Live-bearing fish	Johnson; Zuniga-Vega	Ecology	2009	10.1890/07-1672.1
10	*Catostomus_catostomus*	*Catostomus_catostomus*	Salish sucker	Velez-Espino; Koops	Ecol Model	2012	10.1016/j.ecolmodel.2012.09.022
11	*Catostomus_platyrhynchus_2*	*Catostomus_platyrhynchus*	Mountain sucker	Young; Koops	Fish & Oceans Can	2013	None
12	*Clinocottus_analis*	*Clinocottus_analis*	Woolly sculpin	Davis; Levin	Mar Ecol Prog	2002	10.3354/meps234229

					Series		
13	*Clinocottus_globiceps*	*Clinocottus_globiceps*	Sculpin fish	Pfister	Ecology	1996	10.1007/bf02027951
14	*Clinostomus_elongatus*	*Clinostomus_elongatus*	Redside dace	Velez-Espino; Koops	Ecol Model	2012	10.1016/j.ecolmodel.2012.09.022
15	*Clinostomus_funduloides*	*Clinostomus_funduloides*	Rosyside dace	Peoples	Master Thesis	2010	None
16	*Coregonus_huntsmani*	*Coregonus_huntsmani*	Atlantic whitefish	Velez-Espino; Koops	Ecol Model	2012	10.1016/j.ecolmodel.2012.09.022
17	*Coregonus_reighardi*	*Coregonus_reighardi*	Shortnose cisco	Velez-Espino; Koops	Ecol Model	2012	10.1016/j.ecolmodel.2012.09.022
18	*Coregonus_zenithicus*	*Coregonus_zenithicus*	Shortjaw cisco	Velez-Espino; Koops	Ecol Model	2012	10.1016/j.ecolmodel.2012.09.022
19	*Cottus_bairdi*	*Cottus_bairdi*	Mottled sculpin	Peoples	Master Thesis	2010	None
20	*Cottus_confusus*	*Cottus_confusus*	Shorthead sculpin	Velez-Espino; Koops	Ecol Model	2012	10.1016/j.ecolmodel.2012.09.022
21	*Cottus_sp.*	*Cottus_sp.*	Eastslope sculpin	Velez-Espino; Koops	Ecol Model	2012	10.1016/j.ecolmodel.2012.09.022
22	*Cyprinodon_diabolis_2*	*Cyprinodon_diabolis*	Devils hole pupfish	Beissinger	PeerJ	2014	10.7717/peerj.549

23	*Epinephelus_morio*	*Epinephelus_morio*	Red grouper	Fujiwara; Zhou	Can J Fish Aq Sci	2013	10.1139/cjfas-2012-0520
24	*Erimyzon_sucetta*	*Erimyzon_sucetta*	Lake chubsucker	Velez-Espino; Koops	Ecol Model	2012	10.1016/j.ecolmodel.2012.09.022
25	*Esox_lucius*	*Esox_lucius*	Northern pike	Edeline; Haugen; Weltzien; Claessen; Winfield; Stenseth; Vollestad	Proc R Soc B	2010	10.1098/rspb.2009.1724
26	*Etheostoma_flabellare_2*	*Etheostoma_flabellare*	Fantail darter	Peoples	Master Thesis	2010	None
27	*Gasterosteus_sp.*	*Gasterosteus_sp.*	Enos lake stickleback	Velez-Espino; Koops	Ecol Model	2012	10.1016/j.ecolmodel.2012.09.022
28	*Gasterosteus_sp._2*	*Gasterosteus_sp.*	Misty lake stickleback	Velez-Espino; Koops	Ecol Model	2012	10.1016/j.ecolmodel.2012.09.022
29	*Genypterus_blacodes*	*Genypterus_blacodes*	Pink cusk-eel	Gonzales-Olivares; Aranguiz-Acuna; Ramos-Jiliberto; Rojas-	Fish Res	2009	10.1016/j.fishres.2008.11.006

				Palma			
30	*Genypterus_blacodes_2*	*Genypterus_blacodes*	Pink cusk-eel	Gonzales-Olivares; Aranguiz-Acuna; Ramos-Jiliberto; Rojas-Palma	Fish Res	2009	10.1016/j.fishres.2008.11.006
31	*Huso_huso*	*Huso_huso*	Beluga sturgeon	Doukakis; Babcock; Pikitch; Sharov; Baimukhanov; Erbulekov; Bokova; Nimatov	Cons Biol	2010	10.1111/j.1523-1739.2010.01458.x
32	*Hybognathus_argyritis*	*Hybognathus_argyritis*	Eastern silvery minnow	Velez-Espino; Koops	Ecol Model	2012	10.1016/j.ecolmodel.2012.09.022
33	*Hypseleotris_klunzingeri*	*Hypseleotris_klunzingeri*	Carp gudgeon	Yen; Bond; Shenton; Spring; Mac Nally	J Appl Ecol	2013	10.1111/1365-2664.12074
34	*Lepisosteus_oculatus*	*Lepisosteus_oculatus*	Spotted gar	Velez-Espino;	Ecol Model	2012	10.1016/j.ecolmodel.2012.09.022

				Koops			
35	*Maccullochella_peelii*	*Maccullochella_peelii*	Murray cod	Yen; Bond; Shenton; Spring; Mac Nally	J Appl Ecol	2013	10.1111/1365-2664.12074
36	*Macquaria_ambigua*	*Macquaria_ambigua*	Golden perch	Yen; Bond; Shenton; Spring; Mac Nally	J Appl Ecol	2013	10.1111/1365-2664.12074
37	*Macrhybopsis_storeriana*	*Macrhybopsis_storeriana*	Carmine shiner	Young; Koops	Fish & Oceans Can	2013	None
38	*Melanogrammus_aeglefinus*	*Melanogrammus_aeglefinus*	North Sea haddock	Gerber; Heppell	Cons Biol	2004	10.1016/j.biocon.2004.01.029
39	*Micropterus_dolomieu_3*	*Micropterus_dolomieu*	Smallmouth bass	Sproomberg; Birge	Environ Toxicol Chem	2005	10.1897/04-160.1
40	*Morone_saxatilis*	*Morone_saxatilis*	Striped bass	Gerber; Heppell	Cons Biol	2004	10.1016/j.biocon.2004.01.029
41	*Morone_saxatilis_2*	*Morone_saxatilis*	Striped bass	Velez-Espino; Koops	Ecol Model	2012	10.1016/j.ecolmodel.2012.09.022
42	*Moxostoma_duquesnei*	*Moxostoma_duquesnei*	Black redhorse	Velez-Espino; Koops	Ecol Model	2012	10.1016/j.ecolmodel.2012.09.022
43	*Moxostoma_duquesnei_3*	*Moxostoma_duquesnei*	Black redhorse	Velez-Espino;	Fish & Oceans	2007	None

				Koops	Can		
44	*Moxostoma_hubbsi*	*Moxostoma_hubbsi*	Copper redhorse	Velez-Espino; Koops	Ecol Model	2012	10.1016/j.ecolmodel.2012.09.022
45	*Neogobius_melanostomus_3*	*Neogobius_melanostomus*	Round goby	Sspromberg; Birge	Environ Toxicol Chem	2005	10.1897/04-160.1
46	*Nocomis_leptocephalus*	*Nocomis_leptocephalus*	Bluehead chub	Peoples	Master Thesis	2010	None
47	*Notropis_anogenus*	*Notropis_anogenus*	Pugnose shiner	Velez-Espino; Koops	Ecol Model	2012	10.1016/j.ecolmodel.2012.09.022
48	*Notropis_anogenus_2*	*Notropis_anogenus*	Pugnose shiner	Venturelli; Velez-Espino; Koops	Fish & Oceans Can	2010	None
49	*Notropis_percobromus*	*Notropis_percobromus*	Carmine shiner	Velez-Espino; Koops	Ecol Model	2012	10.1016/j.ecolmodel.2012.09.022
50	*Notropis_percobromus_2*	*Notropis_percobromus*	Silver chub	Young; Koops	Fish & Oceans Can	2013	None
51	*Noturus_stigmosus*	*Noturus_stigmosus*	Northern madtom	Velez-Espino; Koops	Ecol Model	2012	10.1016/j.ecolmodel.2012.09.022
52	*Oligocottus_maculosus*	*Oligocottus_maculosus*	Sculpin fish	Pfister	Ecology	1996	10.1007/bf02027951

53	*Oncorhynchus_clarkii_subsp._lewisi*	*Oncorhynchus_clarkii_subsp._lewisi*	Westslope cutthroat trout	Peterson; Rieman; Young; Brammer	Ecol Appl	2010	10.1890/09-0679.1
54	*Oncorhynchus_clarkii_subsp._lewisi_2*	*Oncorhynchus_clarkii_subsp._lewisi*	Westslope cutthroat trout	Spromberg; Birge	Environ Toxicol Chem	2005	10.1897/04-160.1
55	*Oncorhynchus_kisutch_2*	*Oncorhynchus_kisutch*	Coho salmon	Sprombergg; Birge	Environ Toxicol Chem	2005	10.1897/04-160.1
56	*Oncorhynchus_tshawytscha*	*Oncorhynchus_tshawytscha*	Chinook salmon	Wilson	Cons Biol	2003	10.1046/j.1523-1739.2003.01535.x
57	*Oncorhynchus_tshawytscha_2*	*Oncorhynchus_tshawytscha*	Chinook salmon	Krueger; Rutherford; Mason	Trans Am Fish Soc	2013	10.1080/00028487.2013.797496
58	*Oncorhynchus_tshawytscha_3*	*Oncorhynchus_tshawytscha*	Chinook salmon	Fujiwara; Mohr; Greenberg	PLOS One	2014	10.1371/jourNonel.pone.0085464
59	*Oncorhynchus_tshawytscha_4*	*Oncorhynchus_tshawytscha*	Chinook salmon	Sprombergg; Birge	Environ Toxicol Chem	2005	10.1897/04-160.1
60	*Oncorhynchus_tshawytscha_5*	*Oncorhynchus_tshawytscha_5*	Chinook salmon	Gerber; Heppell	Cons Biol	2004	10.1016/j.biocon.2004.01.029
61	*Osmerus_spectrum*	*Osmerus_spectrum*	Utopia dwarf smelt	Velez-Espino; Koops	Ecol Model	2012	10.1016/j.ecolmodel.2012.09.022

62	*Percina_copelandi*	*Percina_copelandi*	Channel darter	Velez-Espino; Koops	Ecol Model	2012	10.1016/j.ecolmodel.2012.09.022
63	*Percina_copelandi_2*	*Percina_copelandi*	Channel darter	Venturelli; Velez-Espino; Koops	Fish & Oceans Can	2010	None
64	*Phoxinus_oreas*	*Chrosomus_oreas*	Mountain redbelly dace	Peoples	Master Thesis	2010	None
65	*Pimephales_promelas_4*	*Pimephales_promelas*	Fathead minnow	Spromberg; Birge	Environ Toxicol Chem	2005	10.1897/04-160.1
66	*Pimephales_promelas_5*	*Pimephales_promelas_5*	Fathead minnow	Schwindt	PhD Thesis	2013	None
67	*Poecilia_reticulata*	*Poecilia_reticulata*	Trinidadian guppy	Bronikowski; Clark; Rodd; Reznick	Ecology	2002	10.1890/0012-9658(2002)083[2194:PDCOPI]2.0.CO;2
68	*Pterois_miles*	*Pterois_miles*	Common lionfish	Morris; Shertzer; Rice	Biol Invasions	2011	10.1007/s10530-010-9786-8
69	*Pterois_volitans*	*Pterois_volitans*	Red lionfish	Morris; Shertzer; Rice	Biol Invasions	2011	10.1007/s10530-010-9786-8
70	*Pylodictis_olivaris*	*Pylodictis_olivaris*	Flathead catfish	Sakaris; Irwin	Ecol Appl	2010	10.1890/08-0305.1
71	*Retropinna_semoni*	*Retropinna_semoni*	Australian smelt	Yen; Bond;	J Appl Ecol	2013	10.1111/1365-

				Shenton; Spring; Mac Nally			2664.12074
72	*Rhinichthys_cataractae*	*Rhinichthys_cataractae*	Nooksack dace	Velez-Espino; Koops	Ecol Model	2012	10.1016/j.ecolmodel.2012.09.022
73	*Rhinichthys_osculus*	*Rhinichthys_osculus*	Speckled dace	Velez-Espino; Koops	Ecol Model	2012	10.1016/j.ecolmodel.2012.09.022
74	*Salvelinus_confluentus_2*	*Salvelinus_confluentus*	Bull trout	Bowerman	PhD Thesis	2013	None
75	*Salvelinus_fontinalis_subsp._timagamiensis*	*Salvelinus_fontinalis_subsp._timagamiensis*	Aurora Trout	Velez-Espino; Koops	Ecol Model	2012	10.1016/j.ecolmodel.2012.09.022
76	*Salvelinus_malma*	*Salvelinus_malma*	Dolly Varden	Spromberg; Birge	Environ Toxicol Chem	2005	10.1897/04-160.1
77	*Sardina_pilchardus*	*Sardina_pilchardus*	Sardine	Serghini; Boutayeb; Auger; Charouki; Ramzi; Ettahiri; Tchente	Acta Biotheor	2009	10.1007/s10441-009-9090-0
78	*Sprattus_sprattus_subsp._balticus*	*Sprattus_sprattus_subsp._balticus*	European sprat	Haslob; Hauss; Petereit;	Mar Biol	2012	10.1007/s00227-012-1933-6

				Clemmesen; Kraus; Peck			
79	*Stellifer_illecebrosus*	*Stellifer_illecebrosus*	Silver stardrum	Foster; Vincent	Aquat Conserv	2012	10.1002/aqc.2243
80	*Tautogolabrus_adspersus*	*Tautogolabrus_adspersus*	Cunner	Gutjahr-Gobell; Zaroogian; Horowitz; Gleason; Mills	Ecotox & Env Sav	2006	10.1016/j.ecoenv.2005.05.017
81	*Zingel_asper*	*Zingel_asper*	Percid	Labonne; Gaudin	Can J Fish Aq Sci	2006	10.1139/f05-245
82	*Zoarces_viviparus*	*Zoarces_viviparus*	European eelpout	Bergek; Ma; Vetemaa; Franz√©n; Appelberg	Ecotoxicol Environ Safety	2012	10.1016/j.ecoenv.2012.01.019
83	*Plectus_acuminatus*	*Plectus_communis*	Nematode	Kammenga; Van Gestel; Hornung	Ecol Appl	2001	10.2307/3061069
84	*Ambystoma_mexicanum*	*Ambystoma_mexicanum*	Axolotl	Zambrano; Vega; Herrera; Prado; Reynoso	Anim Conserv	2007	10.1111/j.1469-1795.2007.00105.x

85	*Assa_darlingtoni*	*Assa_darlingtoni*	Pouched frog	Keith; Mahony; Hines; Elith; Regan; Baumgartner; Hunter; Heard; Mitchell; Parris; Penman; Scheele; Simpson; Tingley; Tracy; West; Akcakaya	Conserv Biol		2014	10.1111/cobi.12234
86	*Bufo_boreas*	*Anaxyrus_boreas*	Western toad	Biek; Funk; Maxell; Mills	Conserv Biol		2002	10.1046/j.1523-1739.2002.00433.x
87	*Epidalea_calamita_2*	*Epidalea_calamita*	Natterjack toad	Di Minin; Griffiths	Ecography		2011	10.1111/j.1600-0587.2010.06263.x
88	*Geocrinia_alba*	*Geocrinia_alba*	White-bellied frog	Conroy; Brook	Pop Ecol		2003	10.1007/s10144-003-0145-9

89	*Geocrinia_vitellina*	*Geocrinia_vitellina*	Orange-bellied frog	Conroy; Brook	Pop Ecol	2003	10.1007/s10144-003-0145-9
90	*Rana_aurora*	*Rana_aurora*	Red-legged frog	Biek; Funk; Maxell; Mills	Conserv Biol	2002	10.1046/j.1523-1739.2002.00433.x
91	*Rana_catesbeiana*	*Lithobates_catesbeianus*	American bullfrog	Govindarajulu; Altwegg; Anholt	Ecol Appl	2005	10.1890/05-0486
92	*Rana_temporaria*	*Rana_temporaria*	Common frog	Biek; Funk; Maxell; Mills	Conserv Biol	2002	10.1046/j.1523-1739.2002.00433.x
93	*Acropora_hyacinthus*	*Acropora_hyacinthus*	Brush coral	Tanner	J Exp Mar Biol & Ecol	1997	10.1016/S0022-0981(97)00024-5
94	*Agaricia_agaricites*	*Agaricia_agaricites*	Tan lettuce-leaf coral	Hughes; Tanner	Ecology	2000	10.1890/0012-9658(2000)081[2250:RFLHAL]2.0.CO;2
95	*Alcyonium_sp.*	*Alcyonium_sp.*	Dead man's finger coral	McFadden	Ecology	1991	10.2307/1940983
96	*Diploria_strigosa*	*Diploria_strigosa*	Symmetrical brain coral	Edmunds	Mar Ecol Prog Ser	2010	10.3354/meps08595
97	*Goniastrea_aspera*	*Goniastrea_aspera*	Lesser star coral	Babcock	Ecol Monog	1991	10.2307/2937107
98	*Goniastrea_aspera*	*Goniastrea_aspera*	Lesser star coral	Orive	Am Nat	1995	10.1086/285729

	_2						
99	*Goniastrea_favulus*	*Goniastrea_favulus*	Lesser star coral	Babcock	Ecol Monog	1991	10.2307/2937107
100	*Goniastrea_favulus _2*	*Goniastrea_favulus*	Lesser star coral	Orive	Am Nat	1995	10.1086/285729
101	*Leptoseris_cucullat a*	*Helioseris_cucullata*	Sunray lettuce coral	Hughes; Tanner	Ecology	2000	10.1890/0012-9658(2000)081[2250:RFLHAL]2.0.CO;2
102	*Montastrea_annul aris*	*Montastraea_annular is*	Boulder star coral	Hughes; Tanner	Ecology	2000	10.1890/0012-9658(2000)081[2250:RFLHAL]2.0.CO;2
103	*Montastraea_annu laris*	*Montastraea_annular is*	Boulder star coral	Hernandez-Pacheco; Hernandez-Delgado; Sabat	Ecosphere	2011	10.1890/ES10-00065.1
104	*Orbicella_annularis*	*Montastraea_annular is*	Caribbean star coral	Edmunds	Limnol Oceanogr	2015	10.1002/lno.10075
105	*Paramuricea_clava ta*	*Paramuricea_clavata*	Violescent sea-whip; Red gorgonian	Linares; Doak	Mar Ecol Pro Ser	2010	10.3354/meps08437
106	*Paramuricea_clava ta_2*	*Paramuricea_clavata*	Violescent sea-whip; Red gorgonian	Linares; Doak; Coma; Diaz;	Ecology	2007	10.1890/05-1931

				Zabala			
107	*Platygyra_sinensis*	*Platygyra_sinensis*	Lesser valley coral	Babcock	Ecol Monog	1991	10.2307/2937107
108	*Platygyra_sinensis _2*	*Platygyra_sinensis*	Lesser valley coral	Orive	Am Nat	1995	10.1086/285729
109	*Plexaura_A*	*Plexaura_A*	Gorgonian coral	Lasker	Oecologia	1991	10.1007/BF00318 316
110	*Pocillopora_damic ornis*	*Pocillopora_damicorni s*	Cauliflower coral	Tanner	J Exp Mar Biol & Ecol	1997	10.1016/S0022-0981(97)00024-5
111	*Porites_astreoides*	*Porites_astreoides*	Caribbean coral reef	Edmunds	Mar Ecol Prog Ser	2010	10.3354/meps085 95
112	*Platynothrus_peltif er*	*Platynothrus_peltifer*	Oribatid mite	Kammeng a; Van Gestel; Hornung	Ecol Appl	2001	10.2307/3061069
113	*Botrylloides_violac eus*	*Botrylloides_violaceus*	Ascidian	Grey	Oecologia	2011	10.1007/s00442-011-1931-2
114	*Botrylloides_violac eus_2*	*Botrylloides_violaceus*	Star ascidian	Cockrell; Sorte	J Exp Mar Bio & Ecol	2013	10.1016/j.jembe.2 012.11.009
115	*Botryllus_schlosseri*	*Botryllus_schlosseri*	Star ascidian	Cockrell; Sorte	J Exp Mar Bio & Ecol	2013	10.1016/j.jembe.2 012.11.010
116	*Accipiter_cooperii*	*Accipiter_cooperii*	Cooper's hawk	Schumake r; Ernst; White; Baker; Haggerty	Ecol Appl	2004	10.1890/02-5010
117	*Accipiter_gentilis_*	*Accipiter_gentilis*	Northern goshawk	Schumake	Ecol Appl	2004	10.1890/02-5010

	3			r; Ernst; White; Baker; Haggerty				
118	*Agelaius_phoeniceus*	*Agelaius_phoeniceus*	Red-winged blackbird	Blackwell; Huszar; Linz; Dolbeer	J Wildlife Manag		2003	10.2307/3802689
119	*Alcedo_atthis*	*Alcedo_atthis*	European kingfisher	Bunzel; Druke	Lifetime Peproduction in Birds		1989	None
120	*Ammodramus_savannarum*	*Ammodramus_savannarum*	Grasshopper sparrow	Hovick; Miller	Land Ecol		2013	10.1007/s10980-013-9896-7
121	*Amphispiza_belli_subsp._belli*	*Amphispiza_belli_subsp._belli*	Bell's sage sparrow	NA	NA	NA		10.1111/j.1523-1739.2008.01066.x
122	*Anas_fulvigula*	*Anas_fulvigula*	Mottled duck	Rigby; Haukos	Southeast Nat		2014	10.1656/058.013.s505
123	*Anas_laysanensis_2*	*Anas_laysanensis*	Laysan duck	Reynolds; Weiser; Jamieson; Hatfield	J Wild Manag		2013	10.1002/jwmg.582
124	*Anas_platyrhynchos_2*	*Anas_platyrhynchos*	Midcontinental mallard	NA	NA	NA		10.2307/3803153
125	*Anas_platyrhynchos_4*	*Anas_platyrhynchos*	Mallard	Howerter; Anderson;	Wild Monog		2014	10.1002/wmon.1012

				Devries; Joynt; Armstrong ; Emery; Arnold				
126	*Anser_anser*	*Anser_anser*	Greylag goose	NA	NA	NA		10.1163/1570756 10X523260
127	*Anser_caerulescens*	*Chen_caerulescens*	Snow goose	Cooch; Rockwell; Brault	Ecol Monog		2001	10.2307/3100065
128	*Anthropoides_para diseus*	*Anthropoides_paradis eus*	Blue crane	Altwegg; Anderson	Funct Ecol		2009	10.1111/j.1365-2435.2009.01563.x
129	*Bonasa_umbellus*	*Bonasa_umbellus*	Ruffed grouse	NA	NA	NA		10.1002/jwmg.278
130	*Bonasa_umbellus_ 2*	*Bonasa_umbellus*	Ruffed grouse	Tirpak; Giuliano; Miller; Allen; Bittner; Buehler; Edwards; Harper; Igo; Norman; Seamster; Stauffer	Biol Cons		2006	10.1016/j.biocon. 2006.06.014

131	*Bostrychia_hagedash*	*Bostrychia_hagedash*	Hadeda ibis	Duckworth; Altwegg; Harebottle	J Orni		2011	10.1007/s10336-011-0758-2
132	*Bubo_virginianus*	*Bubo_virginianus*	Great horned owl	Schumaker; Ernst; White; Baker; Haggerty	Ecol Appl		2004	10.1890/02-5010
133	*Buteo_jamaicensis*	*Buteo_jamaicensis*	Red-tailed hawk	Schumaker; Ernst; White; Baker; Haggerty	Ecol Appl		2004	10.1890/02-5010
134	*Buteo_lineatus*	*Buteo_lineatus*	Red-shouldered hawk	NA	NA	NA		10.1111/j.1523-1739.2008.01066.x
135	*Buteo_solitarius*	*Buteo_solitarius*	Hawai'ian hawk	Klavitter; Marzluff; Vekasy	J Wild Manage		2003	None
136	*Calidris_temminckii*	*Calidris_temminckii*	Baltic temminck's stint	Koivula; Pakanen; R√∂nk√§; Belda	Avian Biol		2008	10.1111/j.0908-8857.2008.04189.x
137	*Campylorhynchus_brunneicapillus_subsp._sandiegensis*	*Campylorhynchus_brunneicapillus_subsp._sandiegensis*	San Diego cactus wren	Conlisk; Motheral; Chung;	Biol Cons		2014	10.1016/j.biocon.2014.04.010

				Wisinski; Endress				
138	*Centrocercus_urophasianus*	*Centrocercus_urophasianus*	Sage grouse	Johnson	Cons Biol		1999	10.1046/j.1523-1739.1999.97284.x
139	*Certhia_americana*	*Certhia_americana*	Brown Creeper	NA	NA	NA		10.1111/j.1523-1739.2008.01066.x
140	*Ciconia_ciconia_3*	*Ciconia_ciconia*	White stork	Schaub; Pradel; Lebreton	Biol Cons		2004	10.1016/j.biocon.2003.11.002
141	*Cistothorus_palustris*	*Cistothorus_palustris*	Marsh wren	Schumaker; Ernst; White; Baker; Haggerty	Ecol Appl		2004	10.1890/02-5010
142	*Coragyps_atratus*	*Coragyps_atratus*	Black vulture	Blackwell; Avery; Watts; Lowney	J Wild Manage		2007	10.2193/2006-146
143	*Coragyps_atratus_2*	*Coragyps_atratus*	Black vulture	Blackwell; Avery; Watts; Lowney	J Wild Manage		2007	10.2193/2006-146
144	*Dendragapus_obscurus*	*Dendragapus_obscurus*	Blue grouse	Schumaker; Ernst; White;	Ecol Appl		2004	10.1890/02-5010

				Baker; Haggerty			
145	*Dendroica_cerulea*	*Setophaga_cerulea*	Cerulean warbler	Jones; Barg; Sillett; Veit; Robertson	Auk	2004	10.1642/0004-8038(2004)121[0015:MEOSAP]2.0.CO;2
146	*Diomedea_melanophris*	*Thalassarche_melanophris*	Black-browed albatross	Arnold; Brault; Croxall	Ecol Appl	2006	10.1890/03-5340
147	*Dryocopus_pileatus*	*Dryocopus_pileatus*	Pileated woodpecker	Schumaker; Ernst; White; Baker; Haggerty	Ecol Appl	2004	10.1890/02-5010
148	*Falco_naumanni*	*Falco_naumanni*	Lesser kestrel	Hiraldo; Negro; Donazar; Gaona	J Appl Ecol	1996	10.2307/2404688
149	*Falco_peregrinus_3*	*Falco_peregrinus*	Peregrine falcon	Altwegg; Jenkins; Abadi	Ibis	2013	10.1111/ibi.12125
150	*Falco_peregrinus_subsp._anatum*	*Falco_peregrinus_subsp._anatum*	Peregrine falco	Deines; Peterson; Boeckner; Boyle; Keighley;	Ecol Appl	2007	10.1890/06-1090.1

				Kogut; Lubben; Rebarber; Ryan; Tenhumberg; Townley; Tyre			
151	*Forpus_passerinus*	*Forpus_passerinus*	Green-rumped parrotlets	Sandercock; Beissinger	J Appl Stats	2002	10.1080/02664760120108818
152	*Fulmarus_glacialis*	*Fulmarus_glacialis*	Northern fulmar	Kerbiriou; Le Viol; Bonnet; Robert	Popul Ecol	2012	10.1007/s10144-012-0306-9
153	*Gavia_immer*	*Gavia_immer*	Great northern diver	Grear; Meyer; Cooley; Kuhn; Piper; Mitro; Vogel; Taylor; Kenow; Craig; Nacci	J Wild Manag	2010	10.2193/2008-093
154	*Gyps_coprotheres*	*Gyps_coprotheres*	Cape vulture	Monadje	Anim	2013	10.1111/acv.1205

				m; Wolter; Neser; Kane	Conserv			4
155	*Haematopus_ostralegus*	*Haematopus_ostralegus*	Oystercatcher	NA	NA	NA		10.1163/157075609X417143
156	*Haliaeetus_albicilla*	*Haliaeetus_albicilla*	White-tailed eagle	Kruger; Groenkorn; Struwe-Juhl	Biol Cons		2010	10.1016/j.biocon.2009.12.010
157	*Himantopus_novaezelandiae*	*Himantopus_novaezelandiae*	Black stilt	Cruz; Pech; Seddon; Cleland; Nelson; Sanders; Maloney	Biol Cons		2013	10.1016/j.biocon.2013.09.008
158	*Hirundo_rustica_2*	*Hirundo_rustica*	Barn swallow	Gr√ºebler; Korner-Nievergelt; Naef-Daenzer	Ecol & Evol		2014	10.1002/ece3.984
159	*Jynx_torquilla*	*Jynx_torquilla*	Wryneck	Schaub; Reichlin; Abadi; K√©ry; Jenni; Arlettaz	Oecologia		2012	10.1007/s00442-011-2070-5

160	*Lagopus_leucura*	*Lagopus_leucura*	White-tailed ptarmigan	Wilson	BMC Ecol		2012	10.1186/1472-6785-12-9
161	*Lagopus_muta*	*Lagopus_muta*	Rock ptarmigan	Wilson; Martin	BMC Ecol		2012	10.1186/1472-6785-12-9
162	*Lagopus_muta_subsp._japonica*	*Lagopus_muta_subsp._japonica*	Japanese rock ptarmigan	Suzuki; Kobayashi; Nakamura; Takasu	Wildlife Biol		2013	10.2981/13-021
163	*Lichenostomus_melanops_subsp._cassidix*	*Lichenostomus_melanops_subsp._cassidix*	Helmeted honeyeater	McCarthy; Menkhorst; Quin; Smales; Burgman	Species conservation and management: case studies.		2004	10.1111/j.1523-1739.2006.00378.x
164	*Milvus_migrans*	*Milvus_migrans*	Black kite	Sergio; Tavecchia; Blas; Lv≥pez; Tanferna; Hiraldo	Basic Appl Ecol		2011	10.1016/j.baae.2010.11.004
165	*Perisoreus_canadensis*	*Perisoreus_canadensis*	Gray jay	Schumaker; Ernst; White; Baker; Haggerty	Ecol Appl		2004	10.1890/02-5010
166	*Pernis_apivorus_2*	*Pernis_apivorus*	European honey buzzard	Bijlsma; Vermeule	Ardea		2012	10.5253/078.100.0208

				n; Hemerik; Klok			
167	*Phalacrocorax_auritus_2*	*Phalacrocorax_auritus*	Double-crested cormorant	Chastant; King; Weseloh; Moore	J Wild Manag	2014	10.1002/jwmg.628
168	*Phoebastria_immutabilis*	*Phoebastria_immutabilis*	Laysan albatross	Finkelstein; Doak; Nakagawa; Sievert; Klavitter	Anim Conserv	2009	10.1111/j.1469-1795.2009.00311.x
169	*Picoides_arcticus*	*Picoides_arcticus*	Black-backed woodpecker	Rota; Millspaugh; Rumble; Lehman; Kesler	PLOS One	2014	10.1371/journal.pone.0094700
170	*Picoides_borealis*	*Picoides_borealis*	Red-cockaded woodpecker	Maguire; Wilhere; Dong	J Wild Manag	1995	None
171	*Poecile_atricapillus*	*Poecile_atricapillus*	Black-capped chickadee	Schumaker; Ernst; White; Baker; Haggerty	Ecol Appl	2004	10.1890/02-5010
172	*Puffinus_auricularis_subsp._auriculari*	*Puffinus_auricularis_subsp._auricularis*	Townsend's shearwater	Martinez-Gomez;	Biol Cons	2004	None

	s			Jacobsen				
173	*Puffinus_tenuirostris*	*Puffinus_tenuirostris*	Short-tailed shearwater	NA	NA	NA		None
174	*Sterna_antillarum_subsp._browni*	*Sternula_antillarum_subsp._browni*	California least tern	NA	NA	NA		None
175	*Sterna_antillarum_subsp._browni_2*	*Sternula_antillarum_subsp._browni*	California least tern	Ak√ßakaya; Atwood; Breininger; Collins; Duncan	J Wild Manag		2003	10.1111/j.1523-1739.2008.01066.x
176	*Sterna_hirundo*	*Sterna_hirundo*	Common tern	Szostek	J Orni		2011	10.1007/s10336-011-0745-7
177	*Sterna_hirundo_2*	*Sterna_hirundo*	Common tern	Szostek; Schaub; Becker	J Anim Ecol		2014	10.1111/1365-2656.12206
178	*Strix_occidentalis_subsp._caurina*	*Strix_occidentalis_subsp._caurina*	Northern spotted owl	Schumaker; Ernst; White; Baker; Haggerty	Ecol Appl		2004	10.1890/02-5010
179	*Strix_occidentalis_subsp._occidentalis_2*	*Strix_occidentalis_subsp._occidentalis*	California spotted owl	LaHaye; Zimmermann; Gutierrez	Auk		2004	10.1642/0004-8038(2004)121[1056:TVITVR]2.0.CO;2
180	*Sturnella_neglecta*	*Sturnella_neglecta*	Western meadowlark	Schumaker; Ernst; White;	Ecol Appl		2004	10.1890/02-5010

				Baker; Haggerty				
181	*Thalassarche_melanophrys*	*Thalassarche_melanophrys*	Black-browed albatross	Rolland; Nevoux; Barbraud; Weimerskirch	Ecol Appl		2009	10.1890/08-1060.1
182	*Turdus_torquatus*	*Turdus_torquatus*	Ring ouzel	Sim; Rebecca; Ludwig; Grant; Reid	J Anim Ecol		2011	10.1111/j.1365-2656.2010.01750.x
183	*Tympanuchus_cupido*	*Tympanuchus_cupido*	Greater prairie chicken	NA	NA	NA		None
184	*Upupa_epops*	*Upupa_epops*	Hoopoe	Gebreselassie	PhD Thesis		2010	None
185	*Vermivora_chrysoptera*	*Vermivora_chrysoptera*	Golden-winged warbler	Bulluck; Buehler; Vallender; Robertson	Wilson J Ornithol		2013	10.1676/12-154.1
186	*Vireo_latimeri*	*Vireo_latimeri*	Puerto Rican vireo	Woodworth	Cons Biol		1999	10.1046/j.1523-1739.1999.97267.x
187	*Zenaida_macroura*	*Zenaida_macroura*	Mourning dove	Schumaker; Ernst; White; Baker;	Ecol Appl		2004	10.1890/02-5010

				Haggerty			
188	*Adamussium_colbecki*	*Adamussium_colbecki*	Antarctic scallop	Ripley; Caswell	Popul Ecol	2008	10.1007/s10144-008-0075-7
189	*Arctica_islandica*	*Arctica_islandica*	Icelandic cyprine	Ripley; Caswell	Popul Ecol	2008	10.1007/s10144-008-0075-7
190	*Gemma_gemma*	*Gemma_gemma*	Amethyst gem clam	Ripley; Caswell	Popul Ecol	2008	10.1007/s10144-008-0075-7
191	*Geukensia_demissa*	*Geukensia_demissa*	Atlantic ribbed marsh mussel	Ripley; Caswell	Popul Ecol	2008	10.1007/s10144-008-0075-7
192	*Lasaea_rubra*	*Lasaea_rubra*	NA	Ripley; Caswell	Popul Ecol	2008	10.1007/s10144-008-0075-7
193	*Lissarca_miliaris*	*Lissarca_miliaris*	NA	Ripley; Caswell	Popul Ecol	2008	10.1007/s10144-008-0075-7
194	*Lissarca_notorcadensis*	*Lissarca_notorcadensis*	NA	Ripley; Caswell	Popul Ecol	2008	10.1007/s10144-008-0075-7
195	*Mya_arenaria*	*Mya_arenaria*	Soft-shell clam	Brousseau	Mar Biol	1978	10.1007/BF00390542
196	*Mytilus_californianus*	*Mytilus_californianus*	California mussel	Carson; Cook; Lopez-Duarte; Levin	Ecology	2011	10.1890/11-0488.1
197	*Mytilus_galloprovincialis*	*Mytilus_galloprovincialis*	Mediterranean mussel	Carson; Cook; Lopez-Duarte; Levin	Ecology	2011	10.1890/11-0488.1

198	*Nuttallia_obscurata*	*Nuttallia_obscurata*	Varnish clam	Dudas; Dower; Anholt	Ecology	2007	10.1890/06-1216.1
199	*Panope_abrupta*	*Panopea_generosa*	Geoduck	Ripley; Caswell	Popul Ecol	2008	10.1007/s10144-008-0075-7
200	*Tridacna_gigas*	*Tridacna_gigas*	Giant clam	Ripley; Caswell	Popul Ecol	2008	10.1007/s10144-008-0075-7
201	*Yoldia_notabilis*	*Yoldia_notabilis*	NA	Nakaoka	Oikos	1997	10.2307/3546090
202	*Yoldia_notabilis_2*	*Yoldia_notabilis*	NA	Ripley; Caswell	Popul Ecol	2008	10.1007/s10144-008-0075-7
203	*Daphnia_magna*	*Daphnia_magna*	Water flea	Duchet; Coutellec; Franquet; Lagneau; Lagadic	Ecotoxic	2011	10.1007/s10646-010-0507-y
204	*Daphnia_pulex*	*Daphnia_pulex*	Water flea	Duchet; Coutellec; Franquet; Lagneau; Lagadic	Ecotoxic	2010	10.1007/s10646-010-0507-y
205	*Daphnia_pulex_3*	*Daphnia_pulex*	Water flea	Frank; Boll; Kelly	Physiol Zool	1957	None
206	*Lampetra_macrostoma*	*Entosphenus_macrostomus*	Vancouver lamprey	Velez-Espino; Koops	Ecol Model	2012	10.1016/j.ecolmodel.2012.09.022
207	*Lampetra_richardsoni*	*Lampetra_richardsoni*	Morrison lamprey	Velez-Espino;	Ecol Model	2012	10.1016/j.ecolmodel.2012.09.022

				Koops			
208	*Petromyzon_marinus_2*	*Petromyzon_marinus*	Sea lamprey	Velez-Espino; McLaughlin; Pratt	Can J Fish Aq Sci	2008	10.1139/f07-166
209	*Amphimedon_compressa*	*Amphimedon_compressa*	Demosponge	Mercado-Molina; Sabat; Yoshioka	J Exp Mar Biol & Ecol	2011	10.1016/j.jembe.2011.07.018
210	*Spongia_graminea*	*Spongia_graminea*	Gulf grass sponge	Cropper; Di Resta	Ecol Model	1999	10.1016/S0304-3800(99)00039-3
211	*Xestospongia_muta*	*Xestospongia_muta*	Giant barrel sponge	McMurray; Henkel; Pawlik	Ecology	2010	10.3354/meps339093
212	*Polydesmus_angustus*	*Polydesmus_angustus*	West-European millipede	David	Ann Entomol Soc Am	2012	10.1603/AN11151
213	*Strongylocentrotus_franciscanus*	*Mesocentrotus_franciscanus*	Red sea urchin	Ebert; Russell	Mar Ecol Prog Ser	1992	10.3354/meps081031
214	*Isurus_oxyrinchus_2*	*Isurus_oxyrinchus*	Shortfin mako shark	Tsai; Sun; Punt; Liu	ICES J Mar Sci	2014	10.1093/icesjms/fsu056
215	*Haliotis_laevigata*	*Haliotis_laevigata*	Greenlip abalone	Fordham; Mellin; Russell; Akcakaya; Bradshaw; Aiello-	GCB	2013	10.1111/gcb.12289

				Lammens; Caley; Connell; Mayfield; Shepherd; Brook			
216	*Haliotis_rufescens*	*Haliotis_rufescens*	Red abalone	Rogers-Bennett; Leaf	Ecol Appl	2006	10.1890/04-1688
217	*Haliotis_sorenseni*	*Haliotis_sorenseni*	White abalone	Rogers-Bennett; Leaf	Ecol Appl	2006	10.1890/04-1688
218	*Helix_aspersa*	*Cornu_aspersa*	Brown garden snail	Laskowski; Hopkin	Ecotox Environ Safe	1996	10.1006/eesa.1996.0045
219	*Umbonium_costatum*	*Umbonium_costatum*	NA	Noda; Nakao	J Anim Ecol	1996	10.2307/5722
220	*Watersipora_subtorquata*	*Watersipora_subtorquata*	NA	Cockrell; Sorte	J Exp Mar Bio & Ecol	2013	10.1016/j.jembe.2012.11.010
221	*Homo_sapiens_subsp._sapiens*	*Homo_sapiens_sapiens*	Human	Keyfitz; Flieger	Book	1990	0-226-43237-8
222	*Acyrthosiphon_pisum*	*Acyrthosiphon_pisum*	Pea aphid	Hamda; Jevtic; Laskowski	Ecotox	2012	10.1007/s10646-012-0904-5
223	*Acyrthosiphon_pisum_2*	*Acyrthosiphon_pisum*	Pea aphid	Gross; Craig; Hutchinso	Ecology	2002	10.1890/0012-9658(2002)083[3285:BEOADM]2.0.C

				n			O;2
224	*Boloria_eunomia*	*Proclossiana_eunomia*	Bog fritillary	Radchuk; Turlure; Schtickzelle	J Anim Ecol	2012	10.1111/j.1365-2656.2012.02029.x
225	*Cicindela_ohlone*	*Cicindela_ohlone*	Ohlone tiger beetle	Cornelisse; Bennett; Letourneau	PLOS One	2013	10.1371/jourNonel.pone.0071005
226	*Lucanus_miwai*	*Lucanus_miwai*	NA	Huang	J Asia-Pac Entomol	2014	10.1016/j.aspen.2014.03.009
227	*Tribolium_sp.*	*Tribolium_sp.*	Flour beetle	Constantino	Science	1997	None
228	*Ampelisca_abdita*	*Ampelisca_abdita*	Amphipod	Kuhn; Munns; Serbst; Edwards; Cantwell; Gleason; Pelletier; Berry	Env Tox & Chem	2002	10.1002/etc.5620210425
229	*Callinectes_sapidus*	*Callinectes_sapidus*	Blue crab	Miller	Estuaries	2001	10.2307/1353238
230	*Pagurus_longicarpus*	*Pagurus_longicarpus*	Long-clawed hermit crab	Damiani	Ecology	2005	10.1890/04-0956
231	*Palaemonetes_pugio_2*	*Palaemonetes_pugio*	Daggerblade grass shrimp	Sable	PhD Thesis	2007	None
232	*Porcellio_scaber*	*Porcellio_scaber*	Isopod	Kammeng	Ecol Appl	2001	10.2307/3061069

				a; Van Gestel; Hornung			
233	*Acinonyx_jubatus*	*Acinonyx_jubatus*	Cheetah	Crooks; Sanjayan; Doak	Biol Cons	1998	10.1046/j.1523-1739.1998.97054.x
234	*Acinonyx_jubatus_2*	*Acinonyx_jubatus*	Serengeti cheetah	Lubben; Tenhumberg; Tyre; Rebarber	Biol Cons	2008	10.1016/j.biocon.2007.11.003
235	*Aepyceros_melampus*	*Aepyceros_melampus*	Impala	Spinage	Ecology	1972	10.2307/1934778
236	*Ailuropoda_melanoleuca*	*Ailuropoda_melanoleuca*	Giant panda	Carter; Ackleh; Leonard; Wang	Ecol Model	1999	10.1016/S0304-3800(99)00145-3
237	*Alces_alces_6*	*Alces_alces*	Moose	Carroll	MSc Thesis	2013	None
238	*Antechinus_agilis*	*Antechinus_agilis*	Agile antechinus	Lindenmayer; Lacy	Biol Cons	2002	10.1016/S0006-3207(01)00134-3
239	*Bos_gaurus*	*Bos_frontalis*	Gaur	Ahrestani; Iyer; Heitkv∂nig; Prins	Mammal Rev	2011	10.1111/j.1365-2907.2010.00166.x
240	*Brachyteles_hypoxanthus*	*Brachyteles_hypoxanthus*	Northern muriqui	Morris; Altmann; Brockman;	Am Nat	2011	10.1086/657443

				Cords; Fedigan; Pusey; Stoinski; Bronikowski; Alberts; Strier			
241	*Callorhinus_ursinus*	*Callorhinus_ursinus*	Northern fur seal	Barlow; Boneng	Mar Mammal Sci	1991	10.1111/j.1748-7692.1991.tb00550.x
242	*Callospermophilus_lateralis*	*Callospermophilus_lateralis*	Golden-mantled ground squirrel	Hostetler; Kneip; Van Vuren; Oli	PLoS1	2012	10.1371/jourNonel.pone.0034379
243	*Camelus_dromedarius*	*Camelus_dromedarius*	Australian feral camel	Pflaumer	JSM	2013	None
244	*Canis_latrans*	*Canis_latrans*	Coyote	Schumaker; Ernst; White; Baker; Haggerty	Ecol Appl	2004	10.1890/02-5010
245	*Canis_lupus*	*Canis_lupus*	Grey wolf	Chapron; Legendre; Ferriere; Clobert; Haight	C R Biol	2003	10.1016/S1631-0691(03)00148-3
246	*Canis_lupus_3*	*Canis_lupus*	Grey wolf	Miller; Jensen;	Ecol Model	2002	10.1016/S0304-3800(01)00493-8

				Hammill			
247	*Canis_lupus_4*	*Canis_lupus*	Grey wolf	Carroll; Phillips; Schumaker; Smith	Cons Biol	2003	10.1046/j.1523-1739.2003.01552.x
248	*Capra_ibex*	*Capra_ibex*	Alpine ibex	Gaillard; Yoccoz	Ecology	2003	10.1890/02-0409
249	*Castor_canadensis*	*Castor_canadensis*	Beaver	Payne	J Wildlife Manage	1984	10.2307/3808459
250	*Cebus_capucinus*	*Cebus_capucinus*	White faced capuchin monkey	Morris; Altmann; Brockman; Cords; Fedigan; Pusey; Stoinski; Bronikowski; Alberts; Strier	Am Nat	2011	10.1086/657443
251	*Cercopithecus_mitis*	*Cercopithecus_mitis*	Blue monkey	Morris; Altmann; Brockman; Cords; Fedigan; Pusey; Stoinski; Bronikows	Am Nat	2011	10.1086/657443

				ki; Alberts; Strier			
252	*Cervus_elaphus_3*	*Cervus_elaphus*	Red deer	Benton; Grant; Clutton-Brock	Evol Ecol	1995	10.1007/BF01237655
253	*Cervus_elaphus_4*	*Cervus_elaphus*	Elk	Hebblewhite; Merrill	Oikos	2011	10.1111/j.1600-0706.2011.19436.x
254	*Cervus_elaphus_8*	*Cervus_elaphus*	Elk	Raithel; Kaufmann; Pletscher	J Wild Manag	2007	10.2193/2005-608
255	*Clethrionomys_rufocanus*	*Clethrionomys_rufocanus*	Gray-sided vole	Yoccoz; Nakata; Stenseth; Saitoh	Res Popul Ecol	1998	10.1007/BF02765226
256	*Clethrionomys_sp.*	*Clethrionomys_sp.*	Red-backed vole	Row; Wilson; Murray	J Anim Ecol	2014	10.1111/1365-2656.12179
257	*Diceros_bicornis*	*Diceros_bicornis*	Black rhinoceros	Brodie; Muntifering; Hearn; Loutit; Loutit; Brell; Uri-Khob; Leader-	Anim Conserv	2011	10.1111/j.1469-1795.2010.00434.x

				Williams; Preez			
258	*Didelphis_aurita*	*Didelphis_aurita*	Common marsupial	Ferreira; Kajin; Vieira; Zangrandi; Cerqueira; Gentile	Mammal Biol	2013	10.1016/j.mambio.2013.03.002
259	*Dugong_dugon*	*Dugong_dugon*	Dugong	Heinsohn; Lacy; Lindenmayer; Marsh; Kwan; Lawler	Anim Conserv	2004	10.1017/S1367943004001593
260	*Eidolon_helvum*	*Eidolon_helvum*	Straw-colored fruit bats	Hayman; McCrea; Restif; Suu-Ire; Fooks; Wood; Cunningham; Rowcliffe	J Mamm	2012	10.1017/S0950268812000167
261	*Elephas_maximus*	*Elephas_maximus*	Asian elephant	Chelliah; Bukka; Sukumar	Biol Cons	2013	10.1016/j.biocon.2013.05.008

262	*Elephas_maximus_2*	*Elephas_maximus*	Asian elephant	Goswami; Vasudev; Oli	Biol Cons	2014	10.1016/j.biocon.2014.05.026
263	*Enhydra_lutris_2*	*Enhydra_lutris*	Sea otter	Gerber; Tinker; Doak; Estes; Jessup	Ecol Appl	2004	10.1890/03-5006
264	*Eubalaena_glacialis*	*Eubalaena_glacialis*	Northern Right Whale; Northatlantic Right Whale	Fujiwara; Caswell	Ecology	2002	10.2307/3072076
265	*Eumetopias_jubatus*	*Eumetopias_jubatus*	Northern sea lion; Steller sea lion	Holmes; York	Cons Biol	2003	10.1111/j.1523-1739.2003.00191.x
266	*Eumetopias_jubatus_2*	*Eumetopias_jubatus*	Northern sea lion; Steller sea lion	York	Mar Mammal Sci	1994	10.1111/j.1748-7692.1994.tb00388.x
267	*Eumetopias_jubatus_3*	*Eumetopias_jubatus*	Northern sea lion; Steller sea lion	Calkins	Department of Fish and Game	1982	None
268	*Felis_catus*	*Felis_catus*	Feral cat	Budke; Slater	J Appl Anim Welf Sci	2009	10.1080/10888700903163419
269	*Gorilla_beringei*	*Gorilla_beringei*	Mountain gorilla	Morris; Altmann; Brockman;	Am Nat	2011	10.1086/657443

				Cords; Fedigan; Pusey; Stoinski; Bronikowski; Alberts; Strier			
270	*Gulo_gulo*	*Gulo_gulo*	Wolverine	Carroll; Noss; Paquet; Schumaker	Ecol Appl	2003	10.1890/02-5195
271	*Halichoerus_grypus*	*Halichoerus_grypus*	Grey seal	Harwood	J Appl Ecol	1978	10.2307/2402601
272	*Halichoerus_grypus_2*	*Halichoerus_grypus*	Gray Seals	Holma; Lindroos; Oinonen	Nat Res Mod	2014	10.1111/nrm.12034
273	*Hemitragus_jemlahicus*	*Hemitragus_jemlahicus*	Himalayan tahr	Caughley	Ecology	1966	10.2307/1935638
274	*Hippocamelus_bisulcus*	*Hippocamelus_bisulcus*	Huemul deer	Corti; Wittmer; Festa-Bianchet	J Mamm	2010	10.1644/09-MAMM-A-047.1
275	*Hystrix_refossa*	*Hystrix_refossa*	Fossil porcupine	Monchot; Fernandez; Gaillard	J Arch Sci	2012	10.1016/j.jas.2012.04.037
276	*Kobus_ellipsiprymn*	*Kobus_ellipsiprymnus*	Waterbuck	Van Sickle;	J Wildlife	1987	10.2307/3801764

	us			Atwell; Craig	Manage		
277	*Lagothrix_lagothricha*	*Lagothrix_lagotricha*	Humboldt's woolly monkey	Defler	Book	2014	978-1-4939-0697-0
278	*Lemmus_lemmus*	*Lemmus_lemmus*	True lemming	Row; Wilson; Murray	J Anim Ecol	2014	10.1111/1365-2656.12179
279	*Leopardus_pardalis*	*Leopardus_pardalis*	Ocelot	Haines; Tewes; Laack; Grant; Young	Biol Cons	2005	10.1016/j.biocon.2005.06.032
280	*Lepus_americanus*	*Lepus_americanus*	Snowshoe hare	Meslow; Keith	J Wildlife Manage	1968	10.2307/3799557
281	*Lepus_europaeus_3*	*Lepus_europaeus*	European hare	Marboutin; Peroux	J Appl Ecol	1995	10.2307/2404820
282	*Lontra_canadensis*	*Lontra_canadensis*	River otter	Gorman; McMillan; Erb; Deperno; Martin	Am Midl Nat	2008	10.1674/0003-0031(2008)159[98:SACMOA]2.0.CO;2
283	*Loxodonta_africana*	*Loxodonta_africana*	African elephant	Chelliah; Bukka; Sukumar	Biol Cons	2013	10.1016/j.biocon.2013.05.008
284	*Lycaon_pictus*	*Lycaon_pictus*	African wild dog	Cross; Beissinger	Cons Biol	2001	10.1111/j.1523-1739.2001.00031.x

285	*Lynx_canadensis*	*Lynx_canadensis*	Canadian lynx	Row; Wilson; Murray	J Anim Ecol	2014	10.1111/1365-2656.12179
286	*Lynx_rufus*	*Lynx_rufus*	Bobcat	Schumaker; Ernst; White; Baker; Haggerty	Ecol Appl	2004	10.1890/02-5010
287	*Macaca_mulatta*	*Macaca_mulatta*	Rhesus macaque	Hernandez-Pacheco; Rawlins; Kessler; Williams; Ruiz-Maldonado; Gonzalez-Martinez; Ruiz-Lambides; Sabat	Am J Primatol	2013	10.1002/ajp.22177
288	*Macaca_mulatta_2*	*Macaca_mulatta*	Rhesus macaque	Blomquist; Sade; Berard	Int J Primatol	2010	10.1007/s10764-010-9461-z
289	*Macaca_mulatta_3*	*Macaca_mulatta*	Rhesus macaque	Kessler; Pacheco; Rawlings;	Am J Primatol	2014	10.1002/ajp.22323

				Ruiz-Lambrides; Delgado; Sabat			
290	*Macaca_mulatta_4*	*Macaca_mulatta*	Rhesus macaque	Hernandez-Pacheco; Delgado; Rawlins; Kessler; Ruiz-Lambides; Maldonado; Sabat	Am J Primatol	2015	10.1002/ajp.22375
291	*Macropus_eugenii*	*Macropus_eugenii*	Tammar wallaby	Chambers; Bencini	Wildl Res	2010	10.1071/WR10080
292	*Marmota_flaviventris_2*	*Marmota_flaviventris*	Yellow-bellied marmot	Ozgul; Oli; Armitage; Blumstein; Van Vuren	Am Nat	2009	10.1086/597225
293	*Marmota_flaviventris_3*	*Marmota_flaviventris*	Yellow-bellied marmot	Ozgul; Oli; Armitage; Blumstein; Van Vuren	Am Nat	2009	10.1086/597225
294	*Microtus_oeconomus*	*Microtus_oeconomus*	Root vole	Johannesen; Aars; Andreassen; Ims	Popul Ecol	2003	10.1007/s10144-003-0139-7

295	*Microtus_sp.*	*Microtus_sp.*	Field mouse	Row; Wilson; Murray	J Anim Ecol	2014	10.1111/1365-2656.12179
296	*Mirounga_angustirostris*	*Mirounga_angustirostris*	Northern elephant seal	Clinton; Le Boeuf	Ecology	1993	10.2307/1939945
297	*Mirounga_leonina_3*	*Mirounga_leonina*	Southern elephant seal	New; Clark; Costa; Fleishman; Hindell; Klanjsek; Lusseau; Kraus; McMahon; Robinson; Schick; Schwarz; Simmons; Thomas; Tyack; Harwood	Mar Ecol-Prog Ser	2014	10.3354/meps10547
298	*Mustela_erminea*	*Mustela_erminea*	Stoat	Wittmer; Powell; King	J Anim Ecol	2007	10.1111/j.1365-2656.2007.01274.x
299	*Odocoileus_virginianus_3*	*Odocoileus_virginianus*	White-tailed deer	Chitwood; Lashley;	J Wildlife Manage	2015	10.1002/jwmg.835

				Kilgo; Moorman; Deperno			
300	*Odocoileus_virginianus_subsp._borealis*	*Odocoileus_virginianus_subsp._borealis*	White-tailed deer	Jensen	Ecol Model	1995	10.1016/0304-3800(93)E0081-D
301	*Onychogalea_fraenata*	*Onychogalea_fraenata*	Bridled nailtail wallaby	Fisher; Hoyle; Blomberg	Ecol Appl	2000	10.2307/2641054
302	*Orcinus_orca_2*	*Orcinus_orca*	Killer whale	Velez-Espino; Ford; Araujo; Ellis; Parken; Balcomb	Can Tech Report Fish & Aq Sci	2014	978-1-100-23563-9
303	*Oreamnos_americanus*	*Oreamnos_americanus*	Mountain goat	Festa-Bianchet; Urquhart; Smith	Can J Zool	1994	10.1139/z94-004
304	*Ovis_aries_2*	*Ovis_aries*	Soay sheep	Clutton-Brock; Price; Albon; Jewell	J Anim Ecol	1992	10.2307/5330
305	*Ovis_canadensis_4*	*Ovis_canadensis*	Bighorn sheep	Rubin; Boyce;	J Wild Manag	2002	10.2307/3803144

				Caswell-Chen			
306	*Ovis_canadensis_5*	*Ovis_canadensis*	Bighorn sheep	Jorgenson; Festa-Bianchet; Gaillard; Wishart	Ecology	1997	10.1890/0012-9658(1997)078[1019:EOASDA]2.0.CO;2
307	*Ovis_canadensis_subsp._sierrae*	*Ovis_canadensis_subsp._sierrae*	Sierra Nevada bighorn sheep	Johnson; Mills; Wehausen; Stephenson	Ecology	2010	10.1111/j.1365-2664.2010.01846.x
308	*Pan_troglodytes_subsp._schweinfurthii*	*Pan_troglodytes_subsp._schweinfurthii*	Eastern chimpanzee	Morris; Altmann; Brockman; Cords; Fedigan; Pusey; Stoinski; Bronikowski; Alberts; Strier	Am Nat	2011	10.1086/657443
309	*Panthera_pardus*	*Panthera_pardus*	Leopard	Balme; Slotow; Hunter	Biol Cons	2009	10.1016/j.biocon.2009.06.020
310	*Papio_cynocephalu*	*Papio_cynocephalus*	Olive baboon	Morris;	Am Nat	2011	10.1086/657443

	s			Altmann; Brockman; Cords; Fedigan; Pusey; Stoinski; Bronikowski; Alberts; Strier			
311	*Peromyscus_maniculatus_2*	*Peromyscus_maniculatus*	Deer mouse	Tallmon	PhD Thesis	2001	None
312	*Petauroides_volans*	*Petauroides_volans*	Greater glider	Lindenmayer; Lacy; Pope	Ecol Appl	2000	10.2307/2641117
313	*Phacochoerus_aethiopicus*	*Phacochoerus_aethiopicus*	Desert warthog	Rodgers	Mammalia	1984	10.1515/mamm.1984.48.3.327
314	*Phascolarctos_cinereus*	*Phascolarctos_cinereus*	Koala	Baxter	Cons Biol	2006	10.1111/j.1523-1739.2006.00378.x
315	*Phascolarctos_cinereus_2*	*Phascolarctos_cinereus*	Koala	Rhodes; Ng; de Villiers; Preece; McAlpine; Possingham	Biol Cons	2011	10.1016/j.biocon.2010.12.027
316	*Phoca_vitulina_2*	*Phoca_vitulina*	Harbor seal	Heide-	Ambio	1992	10.2307/4314005

				Jorgensen; Hv§rkv∂n en; Aberg			
317	*Phocarctos_hooker i*	*Phocarctos_hookeri*	New Zealand sea lion	Lalas; Bradshaw	Biol Cons	2003	10.1016/S0006-3207(02)00421-4
318	*Pongo_abelii*	*Pongo_abelii*	Sumatran orangutan	Wich; Utami-Atmoko; Setia; Rijksen; Schv°rma nn; van Hooff; van Schaik	J Hum Evol	2004	10.1016/j.jhevol.2 004.08.006
319	*Presbytis_thomasi*	*Presbytis_thomasi*	Thomas's langur	Wich; Steenbeek ; Korstjens; Willems; van Schalk	Am J Primatol	2007	10.1002/ajp.2038 6
320	*Procyon_lotor*	*Procyon_lotor*	Raccoon	Schumake r; Ernst; White; Baker; Haggerty	Ecol Appl	2004	10.1890/02-5010
321	*Propithecus_edwar dsi*	*Propithecus_edwardsi*	Milne-Edwards' sifaka	Dunham; Erhart;	Biol Cons	2008	10.1016/j.biocon. 2007.10.006

				Overdorff; Wright			
322	*Propithecus_verreauxi*	*Propithecus_verreauxi*	Verreaux's sifaka	Morris; Altmann; Brockman; Cords; Fedigan; Pusey; Stoinski; Bronikowski; Alberts; Strier	Am Nat	2011	10.1086/657443
323	*Pseudalopex_culpaeus*	*Lycalopex_culpaeus*	Andean fox	Novaro; Funes; Walker	J Appl Ecol	2005	10.1111/j.1365-2664.2005.01067.x
324	*Puma_concolor_2*	*Puma_concolor*	Cougar	Robinson; Wielgus; Cooley; Cooley	Ecol Appl	2008	10.1890/07-0352.1
325	*Puma_concolor_4*	*Puma_concolor*	Cougar	Wielgus; Morrison; Cooley; Maletzke	Biol Cons	2013	10.1016/j.biocon.2013.07.008
326	*Rangifer_tarandus_2*	*Rangifer_tarandus*	Western Arctic caribou; Reindeer	Haskell; Ballard	J Wild Manag	2007	10.2193/2006-349
327	*Rangifer_tarandus_subsp._tarandus*	*Rangifer_tarandus_subsp._tarandus*	Reindeer	Messier; Huot; Le	Arctic	1988	10.14430/arctic1733

				Henaff; Luttich			
328	*Rattus_fuscipes*	*Rattus_fuscipes*	Bush rat	Lindenmayer; Lacy	Biol Cons	2002	10.1016/S0006-3207(01)00134-3
329	*Saguinus_fuscicollis*	*Saguinus_fuscicollis*	Saddlebacked tamarin	Watsa	PhD Thesis	2013	10.7936/K7DB7ZTD
330	*Saguinus_imperator*	*Saguinus_imperator*	Emperor tamarin	Watsa	PhD Thesis	2013	10.7936/K7DB7ZTD
331	*Saimiri_sciureus*	*Saimiri_sciureus*	Squirrel monkey	Zimbler-Delorenzo; Dobson	Am J Primatol	2011	10.1002/ajp.20970
332	*Sciurus_niger_subsp._cinereus*	*Sciurus_niger_subsp._cinereus*	Delmarva fox squirrel	Hilderbrand; Gardner; Ratnaswamy; Keller	Biol Cons	2007	10.1016/j.biocon.2007.01.015
333	*Sigmodon_hispidus*	*Sigmodon_hispidus*	Hispid cotton rat	Sauer; Slade	J Mamm	1985	10.2307/1381244
334	*Sigmodon_hispidus_2*	*Sigmodon_hispidus*	Hispid cotton rat	Sauer; Slade	J Mamm	1985	10.2307/1381244
335	*Spermophilus_armatus*	*Urocitellus_armatus*	Uinta ground squirrel	Oli; Slade; Dobson	Ecology	2001	10.1890/0012-9658(2001)082[1921:EODROU]2.0.CO;2
336	*Spermophilus_armatus_2*	*Urocitellus_armatus*	Uinta ground squirrel	Oli; Slade; Dobson	Ecology	2001	10.1890/0012-9658(2001)082[1921:EODROU]2.0.C

							O;2
337	*Spermophilus_beldingi*	*Urocitellus_beldingi*	Belding's ground squirrel	Sherman; Morton	Ecology	1984	10.2307/1939140
338	*Spermophilus_columbianus*	*Urocitellus_columbianus*	Columbian ground squirrel	Dobson; Oli	Am Nat	2001	10.1086/321322
339	*Spermophilus_columbianus_3*	*Urocitellus_columbianus*	Columbian ground squirrel	Zammuto	Ecology	1987	10.2307/1939219
340	*Spermophilus_dauricus*	*Spermophilus_dauricus*	Daurian ground squirrel	Luo; Fox	J Mamm	1990	10.2307/1381947
341	*Sus_scrofa_subsp._scrofa*	*Sus_scrofa_subsp._scrofa*	Wild boar	Gamelon; Besnard; Gaillard; Servanty; Baubet; Brandt; Gimenez	Evol	2011	10.1111/j.1558-5646.2011.01366.x
342	*Sus_scrofa_subsp._scrofa_2*	*Sus_scrofa_subsp._scrofa*	Wild boar	Gamelon; Gaillard; Servanty; Gimenez; Toigo; Baubet; Klein; Lebreton	J Appl Ecol	2012	10.1111/j.1365-2664.2012.02160.x
343	*Tamiasciurus_douglasii*	*Tamiasciurus_douglasii*	Douglas squirrel	Schumaker; Ernst; White;	Ecol Appl	2004	10.1890/02-5010

				Baker; Haggerty			
344	*Tamiasciurus_hudsonicus*	*Tamiasciurus_hudsonicus*	American red squirrel	McAdam; Boutin; Sykes; Humphries	EcoSci	2007	10.2980/1195-6860(2007)14[362:LHOFRS]2.0.CO;2
345	*Trichosurus_caninus*	*Trichosurus_caninus*	Mountain brushtail possum	Lindenmayer; Lacy; Pope	Ecol Appl	2000	10.2307/2641117
346	*Urocyon_littoralis_2*	*Urocyon_littoralis*	Island fox	Hudgens; Garcelon	Oecologia	2011	10.1007/s00442-010-1761-7
347	*Ursus_americanus*	*Ursus_americanus*	American black bear	Freedman; Portier; Sunquist	Ecol Model	2003	10.1016/S0304-3800(03)00171-6
348	*Ursus_americanus_2*	*Ursus_americanus*	American black bear	Clark; Eastridge	J Wildlife Manage	2006	10.2193/0022-541X(2006)70[1094:GASOBB]2.0.CO;2
349	*Ursus_americanus_3*	*Ursus_americanus*	American black bear	Hebblewhite; Percy; Serrouya	Biol Cons	2003	10.1016/S0006-3207(02)00341-5
350	*Ursus_americanus_4*	*Ursus_americanus*	American black bear	Mitchell; Pacifici; Grand; Powell	Ursus	2009	10.2192/1537-6176-20.2.77
351	*Ursus_americanus*	*Ursus_americanus*	American black	Lewis;	Ecol	2014	10.1016/j.ecolmo

	_5		bear	Breck; Wilson; Webb	Model		del.2014.08.021
352	*Ursus_americanus_subsp._floridanus*	*Ursus_americanus_subsp._floridanus*	Florida black bear	Hostetler; McCown; Garrison; Neils; Barrett; Sunquist; Simek; Oli	Biol Cons	2009	10.1016/j.biocon.2009.05.029
353	*Ursus_arctos_3*	*Ursus_arctos*	Grizzly bear	Carroll; Noss; Paquet; Schumaker	Ecol Appl	2003	10.1890/02-5195
354	*Ursus_arctos_subsp._horribilis_2*	*Ursus_arctos_subsp._horribilis*	Grizzly bear	Pease; Mattson	Ecology	1999	10.2307/177030
355	*Ursus_arctos_subsp._horribilis_5*	*Ursus_arctos_subsp._horribilis_5*	Grizzly bear	Wielgus	Biol Cons	2002	10.1016/S0006-3207(01)00265-8
356	*Ursus_arctos_subsp._yesoensis*	*Ursus_arctos_subsp._yesoensis*	Japanese brown bear	Kohira; Okada; Nakanishi; Yamanaka	Ursus	2009	10.2192/1537-6176-20.1.12
357	*Ursus_maritimus_2*	*Ursus_maritimus*	Polar bear	Hunter; Caswell; Runge; Regehr;	Ecology	2010	10.1890/09-1641

				Amstrup; Stirling			
358	*Vulpes_vulpes*	*Vulpes_vulpes*	Red fox	McLeod; Saunders	Wildl Res	2001	10.1071/WR00104
359	*Vulpes_vulpes_2*	*Vulpes_vulpes*	Red fox	Schumaker; Ernst; White; Baker; Haggerty	Ecol Appl	2004	10.1890/02-5010
360	*Vulpes_vulpes_3*	*Vulpes_vulpes*	Red fox	Devenish-Nelson; Harris; Soulsbury; Richards; Stephens	Oikos	2013	10.1111/j.1600-0706.2012.20706.x
361	*Zalophus_californianus*	*Zalophus_californianus*	California sea lion	Wielgus; Gonzalez-Suarez; Aurioles-Gamboa; Gerber	Ecol Appl	2008	10.1890/07-0892.1
362	*Arctodiaptomus_salinus*	*Arctodiaptomus_salinus*	NA	Jim√©nez-Melero; Gilbert; Guerrero	Fresh Biol	2013	10.3354/meps10377
363	*Amphiascus_tenuiremis_2*	*Amphiascus_tenuiremis*	NA	Chandler; Cary;	Environ Sci	2004	10.1021/es049654o

				Bejarano; Pender; Ferry	Technol		
364	*Lepeophtheirus_salmonis*	*Lepeophtheirus_salmonis*	Salmon louse	Groner; Gettinby; Stormoen; Revie; Cox	PLOS One	2014	10.1371/journal.pone.0088465
365	*Oithona_hebes*	*Oithona_hebes*	Copepod	Torres-Sorando; Zacarias; Zoppi de Roa; Rodriguez	Ecol Model	2003	10.1016/S0304-3800(02)00355-1
366	*Capitella_sp.*	*Capitella_sp.*	Polychaete	Hansen; Forbes; Forbes	Funct Ecol	1999	10.1046/j.1365-2435.1999.00299.x
367	*Nephtys_incisa*	*Nephtys_incisa*	Polychaete	Zajac; Whitlatch	Mar Ecol Prog Ser	1989	10.3354/meps057089
368	*Stratiodrilus_aeglaphilus*	*Stratiodrilus_aeglaphilus*	Freshwater crayfish	Pardo; Vila; Bustamante	Hydrobio	2008	10.1007/s10750-007-9136-8
369	*Streblospio_benedicti_2*	*Streblospio_benedicti*	Polychaete worm	Levin; Caswell; DePatra; Creed	Ecology	1987	10.2307/1939879
370	*Alligator_mississip*	*Alligator_mississippie*	American alligator	Tucker	Book	2001	978-0-949324-89-

	piensis	*nsis*					4
371	*Apalone_mutica*	*Apalone_mutica*	Smooth softshelled turtle	Zimmer-Shaffer; Briggler; Millspaugh	Chel Cons Biol	2014	10.2744/CCB-1109.1
372	*Apalone_spinifera*	*Apalone_spinifera*	Spiny softshell turtle	Zimmer-Shaffer; Briggler; Millspaugh	Chel Cons Biol	2014	10.2744/CCB-1109.1
373	*Caiman_crocodilus*	*Caiman_crocodilus*	Spectacled caiman	Tucker	Book	2001	978-0-949324-89-4
374	*Caretta_caretta*	*Caretta_caretta*	Loggerhead turtle	Crouse; Crowder; Caswell	Ecology	1987	10.2307/1939225
375	*Chelodina_expansa*	*Chelodina_expansa*	Broad-shelled turtle	Spencer; Thomson	Cons Biol	2005	10.1111/j.1523-1739.2005.00487.x
376	*Chelonia_mydas*	*Chelonia_mydas*	Green sea turtle	Chaloupka	Ecol Model	2002	10.1016/S0304-3800(01)00433-1
377	*Chelydra_serpentina_3*	*Chelydra_serpentina*	Common snapping turtle	Zimmer-Shaffer; Briggler; Millspaugh	Chel Cons Biol	2014	10.2744/CCB-1109.1
378	*Chrysemys_picta_3*	*Chrysemys_picta*	Painted turtle	Mitchell	Herpetol	1988	10.2307/1467026

					Monogr		
379	*Clemmys_guttata*	*Clemmys_guttata*	Spotted turtle	Enneson; Litzgus	Biol Cons	2008	10.1016/j.biocon.2008.04.001
380	*Crocodylus_johnstoni*	*Crocodylus_johnstoni*	Freshwater crocodile	Tucker	Book	2001	978-0-949324-89-4
381	*Crocodylus_niloticus_2*	*Crocodylus_niloticus*	Nile crocodile	Hutton	PhD	1984	None
382	*Cryptophis_nigrescens*	*Cryptophis_nigrescens*	Common small-eyed snake	Webb; Brook; Shine	Ecol Res	2002	10.1046/j.1440-1703.2002.00463.x
383	*Emydura_macquarii*	*Emydura_macquarii*	Macquarie turtle	Spencer; Thomson	Cons Biol	2005	10.1111/j.1523-1739.2005.00487.x
384	*Hoplocephalus_bungaroides*	*Hoplocephalus_bungaroides*	Broad-headed snake	Webb; Brook; Shine	Ecol Res	2002	10.1046/j.1440-1703.2002.00463.x
385	*Kinosternon_integrum*	*Kinosternon_integrum*	Mud turtle	Macip-R√≠os; Brauer-Robleda; Zuniga-Vega; Casas-Andreu	Herp J	2011	None
386	*Lacerta_agilis*	*Lacerta_agilis*	Sand lizard	Berglind	Ecol Bull	2000	10.2307/20113253
387	*Malaclemys_terrap*	*Malaclemys_terrapin*	Diamondback	Crawfod;	J Appl Ecol	2013	10.1111/1365-

	in_2		terrapin	Maerz; Nibbelink; Buhlmann ; Norton			2664.12194
388	*Phrynosoma_cornutum*	*Phrynosoma_cornutum*	Texas horned lizard	Wolf; Hellgren; Schauber; Bogosian III; Kazmaier; Ruthven III; Moody	Popul Ecol	2014	10.1007/s10144-014-0450-5
389	*Podocnemis_expansa*	*Podocnemis_expansa*	Arrau turtle	Mogollones; Rodr√≠guez; Hern√°ndez; Barreto	Chel Cons Biol	2010	10.2744/CCB-0778.1
390	*Sceloporus_arenicolus*	*Sceloporus_arenicolus*	Dunes sagebrush lizard	Ryberg; Hill; Painter; Fitzgerald	Cons Biol	2014	10.1111/cobi.12429
391	*Sceloporus_grammicus*	*Sceloporus_grammicus*	Mesquite lizard	P√©rez-Mendoza	Herpetelogia	2013	10.1655/HERPETOLOGICA-D-12-00038R2
392	*Sceloporus_grammicus_2*	*Sceloporus_grammicus*	Mesquite lizard	Zuniga-Vega;	Can J Zool	2008	10.1139/Z08-124

				Mendez-dela Cruz; Cuellar			
393	*Sceloporus_grammicus_3*	*Sceloporus_grammicus*	Mesquite lizard	Zuniga-Vega; Molina-Zuluaga; Hernandez-Gallegos; Manriquez-Moran; Rodriguez-Romero; Villagran-Santa Cruz; Mendez-de la Cruz	Amphibia-Reptilia	2013	10.1163/15685381-00002843
394	*Sceloporus_mucronatus_subsp._mucronatus*	*Sceloporus_mucronatus_subsp._mucronatus*	NA	Ortega-Leon; Smith; Zuniga-Vega; Mendez-de la Cruz	West N Am Naturalist	2007	10.3398/1527-0904(2007)67[492:GADOOP]2.0.CO;2
395	*Sternotherus_odoratus*	*Sternotherus_odoratus*	Common musk turtle	Mitchell	Herpetol Monogr	1988	10.2307/1467026

396	*Vipera_aspis*	*Vipera_aspis*	Asp viper	Altwegg; Dummermuth; Anholt; Flatt	Oikos	2005	10.1111/j.0030-1299.2001.13723.x
397	*Xenosaurus_grandis*	*Xenosaurus_grandis*	Crevice-dwelling lizard	Zuniga-Vega; Valverde; Rojas-Gonzalez; Lemos-Espinal	Copeia	2007	10.1643/0045-8511(2007)7[324:AOTPDO]2.0.CO;2
398	*Xenosaurus_platyceps*	*Xenosaurus_platyceps*	Flathead knob-scaled lizard	Rojas-Gonzalez; Jones; Zuniga-Vega; Lemos-Espinal	Amphibia-Reptilia	2008	10.1163/156853808784124992
399	*Xenosaurus_sp.*	*Xenosaurus_sp.*	Knob-scaled lizard	Zamora-Abrego; Chang; Zuniga-Vega; Nieto-Montes de Oca; Johnson	Herpet	2010	10.1655/09-005.1

400	*Zootoca_vivipara*	*Zootoca_vivipara*	Common lizard	Mugabo; Perret; Legendre; Le Galliard	J Anim Ecol	2013	10.1111/1365-2656.12109
401	*Caenorhabditis_elegans_2*	*Caenorhabditis_elegans*	NA	Li; ju; Liao; Liao	Ecotoxicology	2014	10.1007/s10646-014-1267-x)
402	*Borrelia_burgdorferi*	*Borrelia_burgdorferi*	Borrelia	Hartemink; Randolph; Davis; Heesterbeek	Am Nat	2008	10.1086/587530

Appendix S6. Funding and extended acknowledgements

We acknowledge the following funding agencies in the support of the COMADRE Matrix Database:

Period	Financial support	Leader/recipient
2011	Max Planck Institute for Demographic Research (Germany)	Roberto Salguero-(
2012	European Social Fund (Europe)	Dave Hodgson & S Townley
2012-2015	University of Maryland Agricultural Experiment Station (USA)	Maile Neel
2013-2014	Max Planck Institute for Demographic Research (Germany)	Roberto Salguero-(& Owen Jones
2014-2016	Australian Research Council (Australia)	Roberto Salguero-(
2014-2017	Natural Environmental Research Council (UK)	David Hodgson
2015-2020	Natural Environmental Research Council (UK)	Roberto Salguero-(

The following is a list of extended acknowledgement to funding agenc supported the time each researcher allocated to the preparation of the manuscript:

R.S.-G. was supported by the Australian Research Council DECRA fellowship and the Max Planck Institute for Demographic Research.

C.R.A. was supported by the Max Planck International Research Network on Aging (MaxNetAging).

D.J.H. was supported by the Natural Environment Research Council, European Social Fund and the University of Exeter.

J.C.-C. was supported by the National Socio-Environmental Synthesis Center.

Y.M.B. was supported by a Marie Curie Reintegration grant.

H.C. was supported by an ERC Advanced Grant 322989 and National Science Foundation Grant DEB-1257545.

Appendix S7. Author contributions

The authorship and authorship order of this manuscript was determined following the ICMJE authorship standards (http://www.icmje.org/recommendations/browse/roles-and-responsibilities/defining-the-role-of-authors-and-contributors.html), aided by the table of contributions (below). The first two positions correspond to the leaders of this project, followed by the digitalization team (in alphabetical order), the online portal host IT, and the core committee members (in mostly alphabetical order, weighted by contributions towards seniority; see Supporting Information Appendix S1). Last authorship position constitutes senior authorship.

The authors below are organized in alphabetical last name order. The definitions of each task are:

- *Project vision and scope*: Founded, developed and/or organized the current staff and database structure.
- *Digitization of data*: Digitization of information into COMADRE Animal Matrix Database v. 1.0.0, previous versions, and any other independent database that has been since integrated into COMADRE.
- *Data error check*: Implementation of protocols for error checking and of “hand” error checking of digitized data.
- *Organization of database*: Set the standards for what variables, with what options and in what ways to archive information in COMADRE.
- *Online portal*: Outlined the organization of the portal, the rules of access and distribution of sub-sites, and oversaw its programming outcomes.

- *R scripts:* Developed *R* scripts to manipulated matrices in COMADRE and ancillary information.
- *Analyses*: Carried out analyses presented for summary statistics in the manuscript.
- *Wrote paper*: Wrote the first full draft of the manuscript, including tables, figures and references, and integrated posterior comments by coauthors.
- *Wrote section*: Wrote one section of the manuscript.
- *Edited paper:* Provided significant comments to the paper, as described by the authorship standards of the ICMJE.
- *Compiled Supporting Information Appendixes*: Organized and wrote the first full draft of the SOM, and integrated posterior comments by coauthors.

Authors	Database role	Project vision and scope	Digitization of data	Data error check	Organization of database	Online portal	*R* scripts	Analyses	Wrote paper	Wrote section	Edited paper	Compiled SIAs	Edited SIAs
Archer	2,3		*	*	*						*		*
Bein	3		*	*							*		
Buckley	2				*						*		
Caswell	2	*			*					*	*		*
Che-Castaldo	2		*	*	*						*		*
de Buhr	3		*	*							*		
Farack	3		*	*							*		
Gottschalk	3		*	*							*		
Hartmann	3		*	*							*		
Henning	3		*	*							*		
Hodgson	2		*		*						*		
Hoppe	3		*	*							*		
Jones	1,2	*		*	*	*	*			*	*		*
Römer	3		*	*							*		
Ruoff	3		*	*									
Salguero-Gómez	1,2	*	*	*	*	*	*	*	*	*	*	*	*
Scheuerlein	2				*						*		*
Sommer	3		*	*							*		
Vaupel	2	*			*						*		
Vieregg	4					*							
Voigt	3		*	*							*		
Wille	3		*	*							*		
Zeh	3		*	*							*		

Values for database role: *1* COMADRE team leader; *2* COMADRE Core Committee member; *3* COMADRE Digitization Team member; *4* IT support. See Supporting Information Appendix S1.

Appendix S8. Supporting information references

Caswell, H. (2001) *Matrix Population Models: Construction, Analysis, and Interpretation*, 2nd edition edn. Sinauer Associates, Inc.

Chamberlain, S. A., & Szöcs, E. (2013). taxize: taxonomic search and retrieval in R. F1000Research, 2.

Cochran, M.E. & Ellner, S. (1992) Simple methods for calculating age-based life-history parameters for stage-structured populations. *Ecological Monographs,* **62,** 345-364.

Meslow, E. C & Keith, L. B. (1968) Demographic parameters of a snowshoe hare population. *Journal of Wildlife Management*, **32**, 812-834.

Metcalf, C. J. E. & Pavard, S. (2007) All paths to fitness lead through demography. *Trends in Ecology & Evolution,* **22,** 563-564.

Morris, W. F. & Doak, D. F. (2002) *Quantitative Conservation Biology: Theory and Practice of Population Viability Analysis*. Sinauer Associates, Sunderland, MA, USA.

Salguero-Gómez, R, Jones, O.R., Archer, C.A., Buckley, Y.M., Che-Castaldo, J., Caswell, C., Scheuerlein, A., Conde, D.A., Baudisch, A., Brinks, E., de Buhr, H., Farack, C., Gottschalk, F., Hartmann, A., Henning, A., Hoppe, G., Römer, G., Runge, J., Ruoff, T., Wille, J., Zeh, S., Vieregg, D., Altwegg, R., Colchero, F., Dong, M., Hodgson, D., de Kroon, H., Lebreton, J-D., Metcalf, C.J.E., Neel, M., Parker, I., Takada, T., Valverde, T., Vélez-Espino, L.A., Wardle, G.M., Franco, M. & Vaupel, J.W. The COMPADRE Plant Matrix Database: an online repository for plant population dynamics. *Journal of Ecology*, **103**, 202-218.

Silvertown, J., Franco, M., Pisanty, I. & Mendoza, A (1993) Comparative plant demography: relative importance of life-cycle components to the finite rate of increase in woody and herbaceous perennials. *Journal of Ecology*, **81**, 465-476.

Stubben, C. & Milligan, B. (2007) Estimating and analyzing demographic models using the popbio package in R. *Journal of Statistical Software,* **22,** 1-23.

Stott, I., Hodgson, D. J. & Townley, S. (2012) popdemo: an R package for population demography using projection matrix analysis. *Methods in Ecology and Evolution,* **3,** 797-802.
